# Structural Aspects of the *Ortho* Chloro- and Fluoro- Substituted Benzoic Acids: Implications on Chemical Properties

**DOI:** 10.3390/molecules25214908

**Published:** 2020-10-23

**Authors:** Gulce Ogruc Ildiz, Rui Fausto

**Affiliations:** 1 Department of Chemistry, University of Coimbra, CQC, P 3004−535 Coimbra, Portugal; rfausto@ci.uc.pt; 2 Department of Physics, Faculty of Sciences and Letters, Atakoy Campus, Istanbul Kultur University, 34156 Bakirkoy, Istanbul, Turkey

**Keywords:** *ortho* chloro- and fluoro- substituted benzoic acids, structural aspects, conformational landscape, crystal features, quantum chemical calculations, isolated-molecule infrared spectroscopy, photochemistry

## Abstract

This article presents a detailed comprehensive investigation of the *ortho* fluoro- and chloro- substituted benzoic acids both, as isolated molecules and in the crystalline phase. Quantum chemical calculations performed within the density functional theory (DFT) formalism are used to investigate the potential energy landscapes of the molecules, taking into special consideration the effects of the interactions between the carboxylic group and the *ortho* halogen substituents, as well as the nature of these later on the structure and properties of the investigated systems. The structures of the relevant conformers of the molecules are discussed in comparative terms, and used to rationalize experimental data obtained for the compounds in the gas phase and isolated in low-temperature inert matrices. The UV-induced photofragmentation reactions of two of the compounds isolated in cryogenic inert matrices were studied as illustrative cases. The structures of the crystals reported previously in the literature are revisited and discussed also in a comparative basis. Particular emphasis is given to the analysis of the intermolecular interactions in the different crystals, using Hirshfeld surface analysis, the CE-B3LYP energy decomposition model and the HOMA index, and to their correlation with thermodynamic data.

## 1. Introduction

Fluoro- and chloro-substituted benzoic acid derivatives have been shown to exhibit relevant practical applications, namely as precursors of agrochemical and pharmaceutical products, food additives and dyes [1]. The fluoro-substituted compounds are considered to be environmentally acceptable alternatives to chlorinated compounds [2], which makes them presently particularly relevant. They have been also used as artificial tracers to investigate flow dynamics in geothermal, hydrothermal and oil well applications to optimize oil recovery [3,4,5]. It was also shown that some bacterial strains use fluorobenzoates as the sole source of carbon and energy [6,7], and the metabolic and catabolic processes involving these compounds have been extensively studied [7,8,9,10,11,12]. Due to its complexing ability, the *ortho*-substituted fluorobenzoic acid (2-fluorobenzoic acid) has been for long used in the quantitative determination of iron in aqueous solution [13]. In its turn, the chloro-substituted benzoic acids are amongst the more versatile precursors or intermediates in the synthesis of pesticides [14]. Diclofenac (2-[2-(2,6-dichloroanilino)phenyl]acetic acid), which is a widespread non-steroid anti-inflammatory drug, is synthesized from 2-chlorobenzoic acid and 2,6-dichloroaniline [15].

From a structural point of view, the *ortho* substituted derivatives are particularly interesting compounds, due to the expected intramolecular interactions between the carboxylic acid moiety and the substituents. This subject has been explored in recent studies on the *ortho* mono-substituted fluoro- and chloro- benzoic acid derivatives [16,17]. For both compounds, the asymmetric suibstitution at the *ortho* positions (in one side of the carboxylic substituent there is a hydrogen atom and in the other a halogen atom) results in dissimilar intramolecular interactions in the different conformers of these molecules, which in turn, determine their distinct chemical behavior [16,17]. The two molecules possess two conformers with the carboxylic group in the *trans* configuration (O=C-O-H dihedral ~180°), which have a considerably higher energy compared to the two related forms having the carboxylic group in the *cis* arrangement (O=C-O-H dihedral ~0°). The *trans*→*cis* conversion for the pairs of *trans*/*cis* related conformers was found to take place at very much different rates, as the stabilizing intramolecular interaction between the *ortho* halogen atom and the carboxylic group in one of the *trans* forms leads to a much higher *trans*→*cis* isomerization barrier compared to that existing in the other *trans* conformer, where such interaction is replaced by a destabilizing interaction between the carboxylic group and the *ortho* hydrogen atom. The experimental observation of these effects was achieved using matrix isolation infrared spectroscopy in cryogenic Ar, N_2_ or Xe matrices. In situ irradiation either by UV [17] (for 2-chlorobenzoic acid) or IR [16] (for 2-fluorobenzoic acid) allowed conversion of the abundant low-energy *cis* conformers into the higher-energy *trans* forms, and the kinetics of the subsequent *trans*→*cis* decay by quantum mechanical tunneling were then estimated and found to correlate with the size of the *trans*→*cis* isomerization barriers [16,17].

The different intrinsic characteristics of the fluorine versus chlorine substituent atoms have also been shown to be major factors in determining the structures of the conformers of the corresponding isolated molecules of the *ortho*-halogen substituted benzoic acids [16,17,18,19]. Likewise, they play a key role in defining other properties of the compounds, as for example relative acidities, cell volumes in crystalline phase, dipole moments, spectroscopic data, and melting points [16,17,18,19,20,21,22,23,24,25,26,27,28,29].

Although, there is a significant amount of information on *ortho* chloro- and fluoro- substituted benzoic acids dispersed in the scientific literature (in particular for the two mono-substituted compounds), no systematic investigation has been reported hitherto that can be used as a comprehensive reference work on this family of compounds. This study aims to fill this gap. Here, a detailed comparative analysis of the potential energy landscapes of the isolated molecules of the *ortho* mono- and di-substituted fluoro- and chloro- benzoic acids, performed by using quantum chemical calculations within the density functional theory (DFT) formalism is presented, taking into special consideration the effects of the interactions between the carboxylic group and the *ortho* halogen substituents, as well as the nature of these later. The structures of the relevant conformers of the molecules are also discussed in comparative terms, and used to rationalize experimental data obtained for the isolated molecules of the compounds, in particular, the compounds in the gas phase and isolated in low-temperature inert matrices. The structures of the crystals reported previously in the literature [20,21,22,23,24,30] are revisited and discussed in a comparative basis. Particular emphasis is given to the analysis of the intermolecular interactions in the different crystals and to their correlation with thermodynamic data.

## 2. Experimental and Computational Methods

The compounds 2-Fluorobenzoic acid and 2-chloro-6-fluorobenzoic acids were obtained from Sigma-Aldrich Portugal (>98% purity) (Sigma-Aldrich, Saint Louis, MO, USA), and additionally purified using the usual freeze-pump-thaw method to eliminate trace volatile impurities. The low temperature matrices were obtained by co-deposition of the matrix-gas (Ar or Xe; purities: N60, N48, respectively) and vapors of the compounds obtained by their sublimation, using a Knudsen cell connected to the cryostat, onto a CsI substrate assembled at the cold (10–20 K) tip of the cryostat (APD Cryogenics, Allentown, PA, USA, model DE-202A). The matrix/solute ratios were ~1000 molar ratio. Both the nozzle and the Knudsen cell were kept at room temperature, which was controlled using a Scientific Instruments, Model 9650-1 temperature controller (accuracy: ±0.1 K).

The infrared spectra for the studied matrix-isolated compounds were obtained in a Thermo Nicolet 6700 FTIR spectrometer (Thermo Fisher Scientific, Waltham, MA, USA), with 0.5 cm^−1^ spectral resolution. A DTGS (deuterated triglycine sulphate) detector, a Globar source, and a KBr beam splitter were used. Irradiations were performed using narrowband tunable UV light provided by a Spectra Physics Quanta-Ray optical parametric oscillator (MOPO-SL) (Spectra Physics, Santa Clara, CA, USA) pumped by a Nd:YAG Spectra Physics Quanta-Ray PRO-230-10 pulsed (10 Hz, 10 ns) laser (Spectra Physics, Santa Clara, CA, USA). The UV beam was introduced into the cryostat through a KBr window. Gas phase infrared spectra were taken from the Coblentz Society infrared spectra database [31].

The DFT/B3LYP [32,33,34] calculations for the isolated molecules were undertaken using Gaussian 09 [35], with the 6-311++G(d,p) basis set [36,37,38]. Potential energy profiles were obtained by scaning the conformationally relevant torsional coordinates and optimizing all remaining structural parameters. The calculated vibrational wavenumbers were scaled by 0.978 (our standard scaling factor for this combination of method and basis set [39,40,41]), mainly to account for the effects of basis set limitations, neglected part of electron correlation and anharmonicity. In the spectra simulation, the bands were represented by convoluting each peak (calculated scaled wavenumber and infrared intensity) with a Lorentzian function with a full-width-at-half-maximum (FWHM) of 2 cm^−1^.

Lattice energies of the crystals (E_lat_) of the studied compounds were computed using the CE-B3LYP model (with the 6-31G(d,p) basis set) [42] using CrystalExplorer17 [43], and related with sublimation enthalpies ΔH_sub_(*T*),

ΔH_sub_(*T*) = (E_el_^g^ + E_trans_^g^ + E_rot_^g^ + E_vib_^g^) − (E_el_^g^ + E_vib_^s^) + *pV* = = (E_el_^g^ − E_el_^s^) + (E_vib_^g^ − E_vib_^s^) + 4R*T* = ΔE_el_ + ΔE_vib_ + 4R*T* = −E_lat_ + ΔE_vib_ + 4R*T* (1)

where ideal gas behavior is assumed, and the superscripts g and s refer to the gas and solid crystalline states. Several approaches have been used to obtain “experimental” benchmark lattice energies by estimating the thermal effects, ΔE_vib_ + 4R*T*, at different levels of sophistication. The most common approximates these two terms by −2R*T*, a result that assumes no difference between gas and crystal intramolecular vibrations, and the intermolecular vibrational energy is at the high-temperature limit of 6R*T*. These and other assumptions underlying this approximation are discussed in detail in several places [44,45,46,47,48]. In the CE-B3LYP calculations, molecules within a radius of 20 Å were considered.

## 3. Results and Discussion

### 3.1. Conformers and Barriers to Internal Rotation

**Figure 1:**
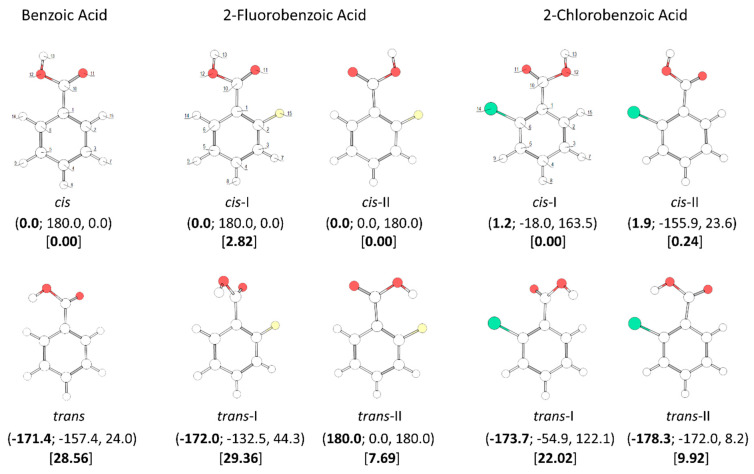
B3LYP/6-311++G(d,p) calculated minimum energy structures of benzoic acid, 2-fluorobenzoic acid and 2-chlorobenzoic acid, with adopted atom numbering. The values of the O=C-O-H, C6-C-C=O and C2-C-C=O dihedral angles (in ^o^) are given in parentheses by this order (the first using bold style). Relative energies are given in square parentheses (in bold), in kJ·mol^−1^. Each depicted non-planar structure has a symmetry-related form. The *cis* benzoic acid conformer is also two-fold degenerate, while the *trans* conformer 4-fold symmetry degenerate.

**Figure 2:**
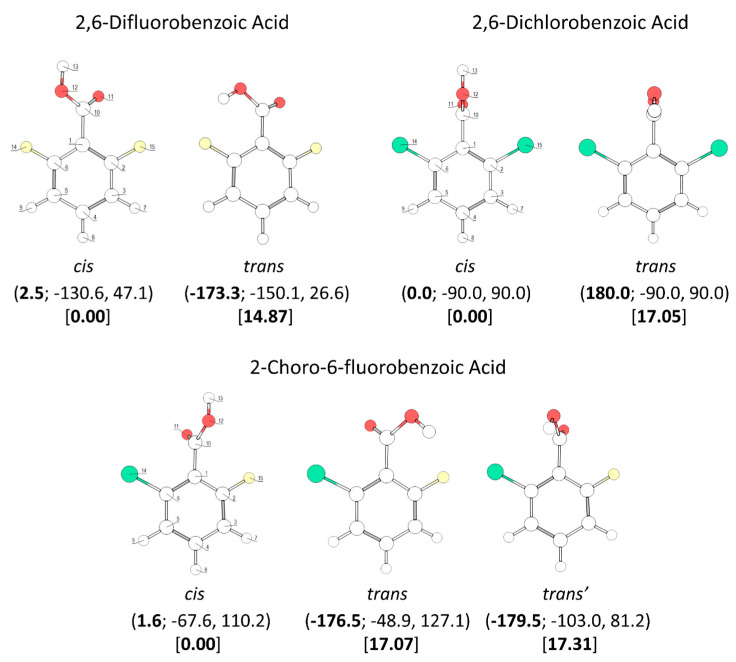
B3LYP/6-311++G(d,p) calculated minimum energy structures of 2,6-difluorobenzoic acid, 2,6-dichlorobenzoic acid and 2-chloro-6-fluorobenzoic acid, with adopted atom numbering. The values of the O=C-O-H, C6-C-C=O and C2-C-C=O dihedral angles (in ^o^) are given in parentheses by this order (the first in bold style). Relative energies are given in square parentheses (in bold), in kJ·mol^−1^. Each conformer of 2,6-dichlorobenzoic acid and 2-chloro-6-fluorobenzoic acid has a symmetry-related identical form; the conformers of 2,6-difluorobenzoic acid have 4 symmetry-equivalent structures.

A throughout conformational search on the B3LYP/6-311++G(d,p) potential energy surfaces of the studied molecules (the mono- and di- *ortho* fluoro- or/and chloro- substituted benzoic acids, plus the parent benzoic acid, for completeness) was undertaken. The identified conformers are presented in Figure 1 and Figure 2.

All studied molecules have two conformationally relevant degrees of freedom, corresponding to the internal rotations around the exocyclic C-C and C-O bonds. For the last, two minimum energy structures exist, which maximize the π-electron delocalization within the carboxylic moiety: the intrinsically most stable *cis* configuration (O=C-O-H dihedral equal to ~0°), and the higher-energy *trans* arrangement (O=C-O-H dihedral equal to ~180°). The reasons for the usual higher stability of the *cis* carboxylic acid structure have been discussed in details elsewhere [49,50,51]. The major reason is the existence of more favorable bond-dipole/bond-dipole interactions in the *cis* structure, where the dipoles associated to the C=O and O-H bonds are nearly anti-paralellely aligned, compared to those existing in the *trans* structure, where these bond-dipoles are approximately parallel. For each carboxylic acid stable configuration (*cis* or *trans*), the number and type of the minimum energy structures differing in the orientation of the carboxylic acid moiety relatively to the aromatic ring (i.e., differing from each other by internal rotation about the exocyclic C-C bond) could be anticipated to be very much dependent on the substitution pattern in the aromatic ring. The obtained results plently confirmed these expectations, and stressed the structural relevance of the intramolecular interactions between the *ortho* substituents and the carboxylic group as well as of the nature of the substituents (H vs. F vs. Cl).

In the case of the parent compound, **benzoic acid** (BA), two conformers exist (Figure 1). The lower-energy *cis* conformer is planar and has two equivalent-by-symmetry forms, corresponding to the experimentally observed species [52,53,54,55,56]. On the other hand, the *trans* conformer has a non-planar structure and has a predicted energy 28.56 kJ·mol^−1^ above that of the *cis* conformer. In the *cis* conformer, the *ortho* hydrogen atoms participate in stabilizing attractive interactions with the oxygen atoms of the carboxylic group (carbonyl, O=, and acid, OH), which favor the planarity of the molecule, while in the *trans* conformer the repulsion between the carboxylic hydrogen atom and the nearby located ring *ortho* hydrogen atom leads to the observed tilt of the carboxylic noiety out of the plane of the ring. Due to its non-planarity and the symmetric substitution in the ring, the *trans* BA conformer has four symmetry equivalent forms. The calculated O=C-O-H angle in this conformer is −171.4° (taking as reference the structure shown in Figure 1) and the two C-C-C=O dihedrals are −157.4° and 24.0°. To the best of our knowledge, *trans* BA has never been experimentally observed (we will return to this point later on in this article).

**Figure 3:**
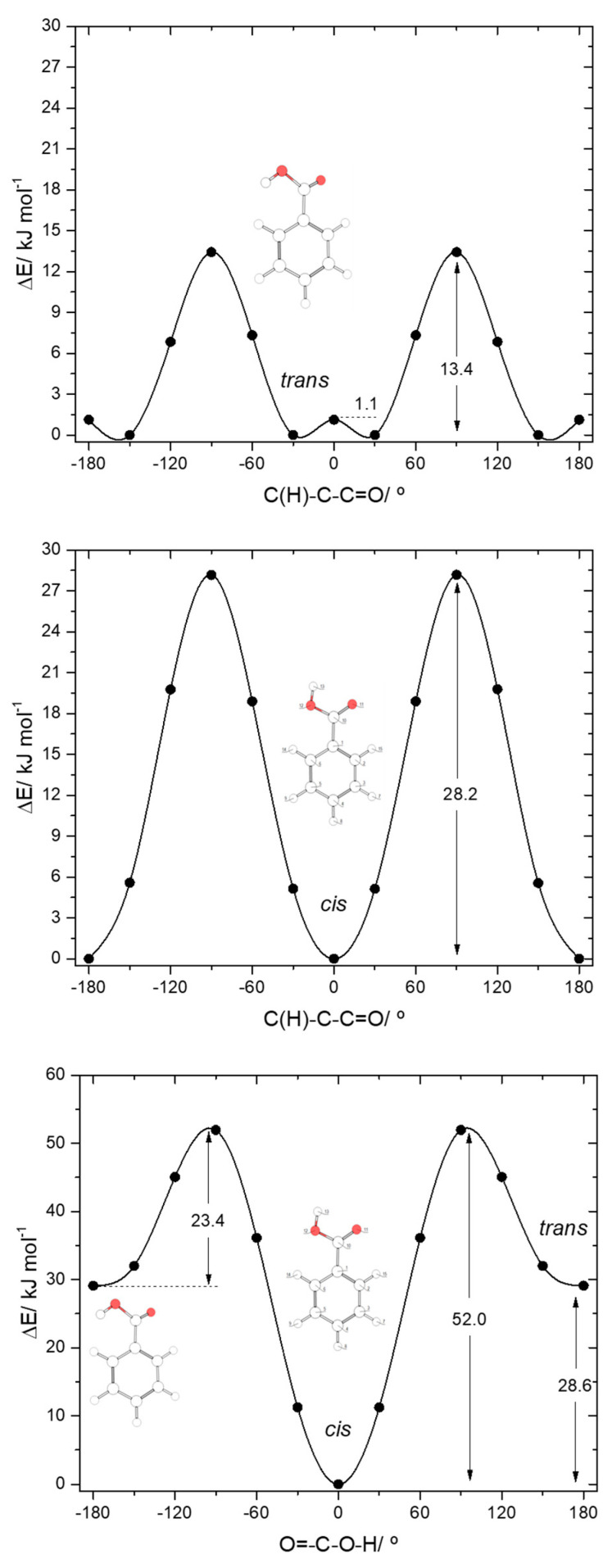
B3LYP/6-311++G(d,p) calculated potential energy profiles for internal rotation about the exocyclic C-C (for *cis* and *trans* arrangement of the carboxylic group) (*top and middle panels*) and about the C-O bond *(bottom panel)* for benzoic acid.

The calculated potential energy profiles for interconversion between the two conformers of BA (internal rotation around the C-O bond) and for internal rotation about the exocyclic C-C bond in the *cis* and *trans* conformers are shown in Figure 3. The *trans*→*cis* barrier is predicted as 23.4 kJ·mol^−1^ (52.0 kJ·mol^−1^ in the reverse direction), with the transition state corresponding to a structure where the carboxylic hydrogen atom is nearly perpendicular to the molecular plane. At the geometry of the transition state, conjugation in the carboxylic fragment is minimal, justifying its high energy.

The profiles for internal rotation around the exocyclic C-C bond are considerably different for the two arrangements of the carboxylic group. For the *cis* arrangement, the energy barrier (28.2 kJ·mol^−1^) is more than twice larger than for the *trans* arrangement (13.4 kJ·mol^−1^). In the *cis* form, the high energy of the transition state for rotation around the C-C bond is a consequence of the break down of the abovementioned stabilizing interactions between the ring *ortho* hydrogen atoms and the oxygen atoms of the carboxylic acid substituent (that operate in the minimum energy structure), as well as the lack of conjugation between the carboxylic substituent and the aromatic ring. This latter effect can be noticed, for example, by looking to the exocyclic C-C bond lengths in the *cis* conformer and in the transition state, respectively 1.486 and 1.500 Å. In the equivalent transition state for the *trans* carboxylic group geometry, there is a favorable interaction between the π–system of the aromatic ring and the O-H carboxylic group (O-H^…^π), which considerably lowers the energy barrier for rotation about the C-C bond. This type of O-H^…^π stabilizing interactions (and similars) has been described for other, structurally related systems [57].

The four symmetry-equivalent *trans* forms can be grouped in two pairs, whose members are separated from each other by a small energy barrier (1.1 kJ·mol^−1^) at the planar transition state geometry (see Figure 3, top panel). Although low, this barrier is still above the zero-point level for the τC-O torsion in the *trans* conformer (calculated as 0.4 kJ·mol^−1^), so that the non-planar structures do indeed correspond to potentially experimentally observable minima.

Both **2-fluorobenzoic acid** (2FBA) and **2-chlorobenzoic acid** (2CBA) have two low-energy *cis* conformers and two higher-energy *trans* conformers (see Figure 1). In the fluoro-substituted compound, three conformers are planar, the two *cis* conformers (*cis*-I and *cis-*II) and one *trans* conformer (*trans*-II), all being unique structures. The additional *trans* conformer (*trans*-I) is non planar, being two-fold degenerate by symmetry. The two *cis* conformers have rather similar energies, with *cis*-II (where the fluoro-substituent stays the same side of the molecule as the acid oxygen atom and a C-H^…^O= stabilizing interaction exists), being the most stable form. The *cis*-I conformer has a relative energy of 2.82 kJ·mol^−1^. In this conformer, the C-H^…^O= interaction present in *cis*-II is replaced by a weaker stabilizing C-H^…^OH interaction. The C-H^…^O= interaction is more efficient in stabilizing *cis*-II than the C-H^…^OH interaction in stabilizing *cis*-I due to the more favorable localization of the interacting carbonyl oxygen lone electron pair, which stays in the molecular plane, whereas in *cis*-I both lone electron pairs of the acid oxygen atom are out of the molecular plane. On the other hand, the interactions between the fluoro-substituent and the oxygen atoms, which are of repulsive nature, also favor a lower energy for *cis*-II compared to *cis*-I. This can be rationalized in similar terms as for the interactions involving the *ortho* hydrogen atom discussed above. In *cis*-I, the F^…^O= repulsion is stronger than the F^…^OH repulsion in *cis*-II because the interacting lone electron pair of the carbonyl oxygen atom is in the same plane as the fluorine atom in the former conformer, while the lone electron pairs of the acid oxygen atom in *cis*-II are both out of the plane in relation to the fluoro-substituent. The calculated atomic charges (Mulliken charges) for the two oxygen atoms, and for the *ortho* fluorine and hydrogen atoms also point to a stronger repulsive F^…^O interaction in *cis*-I and to a stronger attractive H^…^O interaction in *cis*-II. The charges (in units of *e*) of the interacting pairs of atoms are: +0.213(H)/−0.193(OH) and −0.133(F)/−0.293(O=) in *cis*-I, and +0.206(H)/−0.295(O=) and −0.139(F)/−0.196(OH) in the most stable *cis*-II conformer.

The planar *trans* 2FBA conformer (*trans*-II) is stabilized by a O-H^…^F intramolecular hydrogen bond, and has a relative energy of 7.69 kJ·mol^−1^. This energy can be compared with that of the second *trans* 2FBA conformer (*trans*-I), where no such stabilizing interaction exists, which amounts to 29.36 kJ·mol^−1^, very similar to the energy difference between the *trans* and *cis* conformers of benzoic acid (28.56 kJ·mol^−1^; see above). Like for the *trans* BA conformer, in the *trans*-I conformer of 2FBA the OH^…^H repulsive interaction dominates, being the main factor determining the high relative energy and the non-planaritry of the conformer (the C(F)-C-C=O and C(H)-C-C=O angles in *trans*-I are 44.3° and −132.5°, respectively, for the structure shown in Figure 1, which has a symmetry equivalent form where these angles are −44.3° and 132.5°).

**Figure 4:**
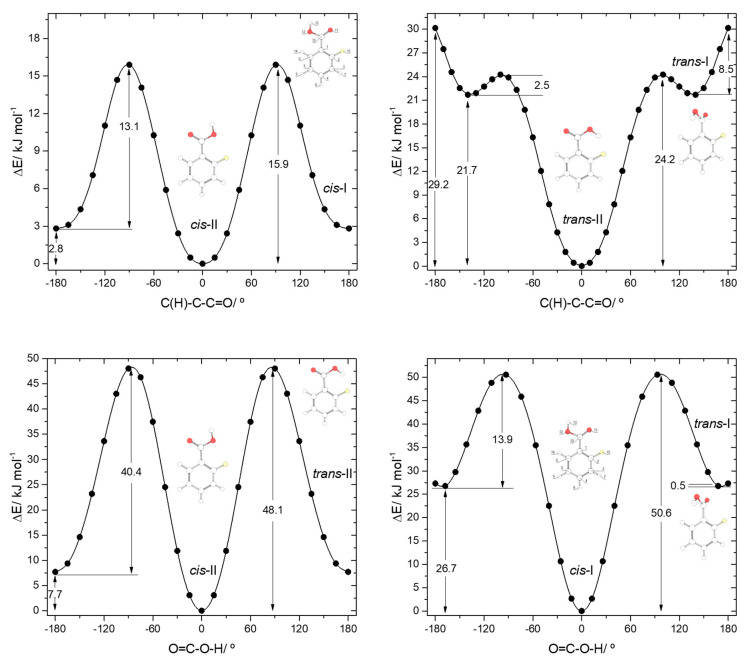
B3LYP/6-311++G(d,p) calculated potential energy profiles for internal rotation about the exocyclic C-C (for *cis* and *trans* arrangement of the carboxylic group) (*top left and top right panels*) and about the C-O bond (connecting the two pairs of conformers, *cis*-II/*trans*-II and *cis*-I/*trans*-I; *bottom left and bottom right panels*) for 2-fluorobenzoic acid.

The potential energy profiles for conversion between the pairs of related *cis* and *trans* 2FBA conformers (*cis*-I and *trans*-I; *cis*-II and *trans*-II) and those for internal rotation about the exocyclic C-C bond in keeping the conformation of the carboxylic acid fragment as *cis* or *trans* are represented in Figure 4. The *trans*-II→*cis*-II conversion takes place over a barrier of 40.4 kJ·mol^−1^ (48.1 kJ·mol^−1^ in the reverse direction), which is more than 3 times higher than that converting *trans*-I into *cis*-I (13.9 kJ·mol^−1^; 50.6 kJ·mol^−1^ in the reverse direction) due to the strong stabilization of *trans*-II originated by the O-H^…^F hydrogen bond.

The internal rotation about the C-C bond for the *cis* carboxylic acid conformation interconverts *cis*-I and *cis*-II and has an associated energy barrier of 13.1 kJ·mol^−1^ in the *cis*-I→*cis*-II direction (15.9 kJ·mol^−1^ in the reverse direction). Interestingly, this barrier is considerably lower than the equivalent one in benzoic acid (28.2 kJ·mol^−1^). Since the intramolecular interactions at the transition states for the C-C internal rotation of both molecules can be expected to be very similar when the carboxylic acid group is in the *cis* conformation, the lower barrier observed in 2FBA compared to BA demonstrates that, globally, the *cis* conformers of 2FBA are destabilized compared to the *cis* form of BA, i.e., F^…^O repulsions dominate over H^…^O attractions.

The interconversion pathway between the two *trans* 2FBA is strikingly affected by the presence of the O-H^…^F hydrogen bond in *trans*-II. Indeed, when the conformation of the carboxylic group is *trans*, the potential energy profile for internal rotation about the C-C bond in 2FBA strongly differs from that of benzoic acid (compare Figure 3, top panel, with Figure 4, top-right panel). The stabilization of *trans*-II, due to the intramolecular H-bond makes the energy barrier for its conversion into the *trans*-I form (24.2 kJ·mol^−1^) to be almost twice the equivalent barrier in *trans* BA (13.4 kJ mol^−1^), while the barrier for the reverse reaction reflects the dominance of strong repulsive interactions in *trans*-I 2FBA, amounting only to 2.5 kJ·mol^−1^. The conversion between the two symmetry-equivalent *trans*-I forms has an associated barrier of 8.5 kJ·mol^−1^, at the planar transition state. Note, also that the *trans*-I→*trans*-II barrier is small, but still considerably above the zero-point energy level of the τC-C torsional vibration in *trans*-I, so that the non-planar *trans*-I conformer should in principle be observable experimentally. We will return to this point later on in this article.

In the case of 2-chlorobenzoic acid, all minimum energy structures are predicted by the calculations as being non-planar. Compared to 2FBA, the order of energy of the two *cis* conformers is predicted by the calculations to be the opposite, with *cis*-I being the most stable conformer. In spite of the small energy difference between the two *cis* conformers predicted by the calculations (0.24 kJ·mol^−1^), their relative order of stability is predicted correctly. In fact, previously reported infrared spectroscopy experiments for the compound isolated in a low temperature argon matrix [17] have unequivocally demonstrated that the *cis*-I form is the lowest energy conformer of 2CBA. The different order of stability of the two *cis* conformers in 2FBA and 2CBA can be rationalized taking into account that the Cl^…^O repulsions are considerably stronger in 2CBA than in 2FBA and that, contrarily to what happens for this latter molecule (see above), in 2CBA, due to the large volume of the chlorine atom, the repulsive Cl^…^O interactions are not significantly affected by the spatial location of the interacting lone electron pair(s) of the oxygen atoms, i.e., the Cl^…^O interaction is as much effective when involving the “in-plane” lone eletron pair of the carbonyl O= atom or the “out-of-plane” lone electron pairs of the acid oxygen atom. In fact, under these assumptions it can even be expected the repulsion to be slightly more important when involving the acid oxygen atom than the carbonyl one (in agreement with the predicted and experimentally observed order of energies of the two conformers of 2CBA), since it is well-known that an acid oxygen atom has a larger effective volume than a carbonyl oxygen [49,50,51].

**Figure 5:**
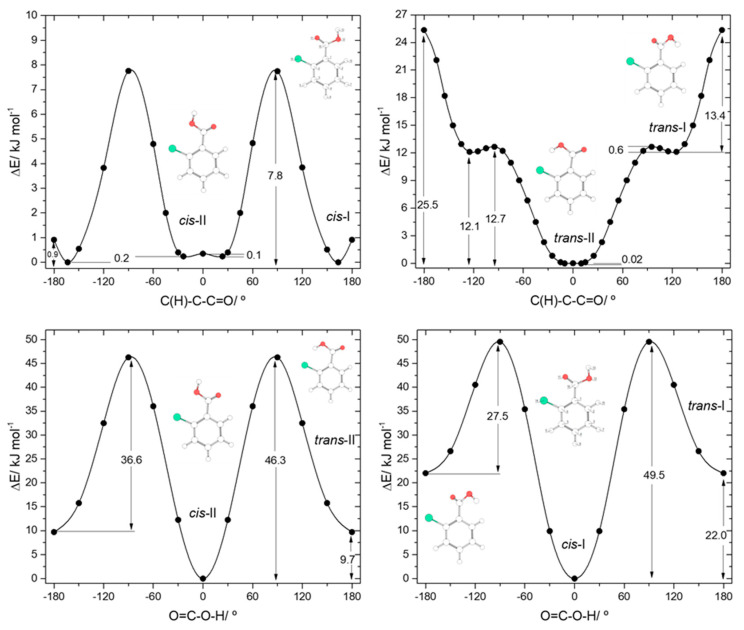
B3LYP/6-311++G(d,p) calculated potential energy profiles for internal rotation about the exocyclic C-C (for *cis* and *trans* arrangement of the carboxylic group) (*top left and top right panels*) and about the C-O bond (connecting the two pairs of conformers, *cis*-II/*trans*-II and *cis*-I/*trans*-I; *bottom left and bottom right panels*) for 2-chlorobenzoic acid.

It should also be noticed that the analysis of the potential energy profile for rotation around the exocyclic C-C bond for the *cis* conformation of the carboxylic group in 2CBA (see Figure 5, top-left panel) reveals that the two-equivalent-by-symmetry *cis*-I forms and the two symmetry-equivalent *cis*-II forms are separated by low energy barriers, at planar transition states. For *cis*-I, the energy barrier (0.9 kJ·mol^−1^) is above the zero-point energy associated with the torsion around the C-C bond (0.2 kJ·mol^−1^), showing that the non-planar structures correspond to experimentally observable species. On the contrary, the energy barrier separating the two *cis*-II minima (0.1 kJ·mol^−1^) is below their zero-point torsional level (0.2 kJ·mol^−1^), so that the experimentally relevant structure of conformer *cis*-II shall be the planar form. According to these conclusions, the gas phase population of the *cis*-I conformer could be expected to be approximately twice that of the *cis*-II conformer. Such result is confirmed by the experimental data reported in [17]. The ratio of the intensities of the infrared bands assigned to these two conformers (*cis*-I:*cis*-II) measured immediately after deposition of an argon matrix of 2CBA was indeed found to be ~2:1.

The main structural characteristics of the *trans* conformers of 2CBA generically follow those described above for the *trans* conformers of 2FBA. Although, two symmetry equivalent minima, corresponding to non-planar geometries, were predicted for the lower energy *trans*-II conformer, the energy barrier separating these minima is lower than 0.1 kJ·mol^−1^ (see Figure 5, top-right panel) and stays below the τC-C zero point level of this conformer (slightly above 0.1 kJ·mol^−1^), so that the planar structure is the experimentally relevant species. Then, like for 2FBA, the lower energy *trans* conformer in 2CBA is a planar unique structure. Moreover, it is also stabilized by an intramolecular hydrogen bond (O-H^…^Cl, in this case). The stabilization of *trans*-II by this interaction is, as it could be anticipated considering the relative strengths of typical O-H^…^Cl and O-H^…^F hydrogen bonds, not as large as in the case of the equivalent conformer of 2FBA: the relative energy of *trans*-II in 2FBA is 7.69 kJ·mol^−1^; in 2CBA, *trans*-II has a relative energy of 9.7 kJ·mol^−1^. The higher-energy *trans*-I conformer has a relative energy of 22.02 kJ·mol^−1^ and is structurally similar to its counterpart in 2FBA (the angles between the planes of the ring and of the carboxylic group are 44.3°, in 2CBA, and 54.9°, in 2FBA). This conformer can be converted into conformer *trans*-II by rotation around the exocyclic C-C bond (Figure 5, top-right panel), the energy barrier for this process being only 0.6 kJ·mol^−1^, but still above the τC-C zero-point level of *trans*-I (~0.3 kJ·mol^−1^). The barrier for the reverse transformation (*trans*-II→*trans*-I) is 12.7 kJ·mol^−1^, which is about half of the equivalent one in 2FBA (21.7 kJ·mol^−1^) and is consistent with the relative strengths of the distinct intramolecular hydrogen bonds present in the *trans*-II conformers of the two molecules.

The potential energy profiles for interconversion between the *cis* and *trans* conformers (Figure 5, bottom panels) follow the trends, already mentioned above, when comparing the *cis*↔*trans* potential energy profiles of 2FBA with the *cis*↔*trans* one in benzoic acid. The *cis*-II→*trans*-II and *cis*-I→*trans*-I transformations all have similar energy barriers (52.0 kJ·mol^−1^ in BA, 48.1 and 50.6 kJ·mol^−1^ in 2FBA, 46.3 and 49.5 kJ·mol^−1^ in 2CBA), but the barriers for the reverse reactions involving the hydrogen bonded conformers (*trans*-II) in 2FBA and 2CBA show the stabilization effect of this intramolecular interaction, increasing along the series BA > 2CBA > 2FBA (23.4, 36.6, 40.4 kJ·mol^−1^, respectively).

It is interesting to examine the available experimental data on benzoic acid, 2FBA and 2CBA in relation to conformational isomerization at light of the above discussed potential energy landscapes. For the three compounds, only the lower energy *cis* conformers were observed in low temperature infrared matrix isolation experiments [16,17,54,55]. The *cis* conformer of BA was also observed in the gas phase both by electron diffraction experiments and microwave spectroscopy [52,53], and the gas phase infrared spectrum of this species has also been reported [56]. On the other hand, the *trans* conformer of BA has never been observed experimentally. In the case of 2FBA, the intramolecularly hydrogen bonded conformer of 2FBA (*trans*-II) was detected in gas phase (together with the two *cis* conformers) by microwave spectroscopy [18], and it has been produced in argon and N_2_ cryomatrices as result of vibrational excitation of the *cis* conformers (using infrared narrowband in situ irradiation) [16]. The existence of the highest energy *trans*-I conformer of 2FBA was inferred indirectly in the matrix isolation conformational studies reported in Ref. [16], but it could not be directly observed due to its fast spontaneous conversion to the lowest-energy *cis* conformers. For 2CBA, in situ UV irradiation of the matrix-isolated *cis* conformers was shown to lead to their conversion into their *trans* counterparts, which were then found to decay by quantum mechanical tunneling back to the *cis* forms [17]. In Ref. [17], the intramolecularly hydrogen bonded *trans*-II conformer was directly observed, while the highest-energy *trans*-I conformer was only possible to observe experimentally upon OH→OD isotopic substitution, in order to make its tunneling conversion into the *cis* form slow enough.

The experimental observation of the *cis* forms was expected for all molecules, considering their low energies (see Figure 1) and the fact that they correspond to deep minima in the potential energy surfaces. The intramolecularly hydrogen bonded *trans*-II conformer of 2FBA is also a well-defined minimum with a high energy barrier (40.4 kJ·mol^−1^) of conversion into *cis*-II (see Figure 4, bottom-left panel), and has an expected population in the gas phase at room temperature of ca. 2% [16], justifying its experimental observation in the microwave experiment [18]. *Trans*-II was not detected in the deposited cryogenic matrices [16] most probably because its expected population in the matrix is below the sensitivity of the technique (the infrared spectra is complex, showing the vibrational signatures of the two *cis* conformers and the spectra of *trans*-II conformer is not much different from those of the *cis* forms, which complicates its experimental detection by this technique). However, once *trans*-II is produced in situ by vibrational excitation (upon infrared irradiation) of the matrix-isolated conformer *cis*-II, the high energy barrier for the *trans*-II→*cis*-II conversion precludes this transformation to take place (the over the barrier thermal process is not possible at all, and the experiments showed that quantum mechanical tunneling is also inneficient in this case). It can then be concluded that the *trans*-II conformer of 2FBA is a stable species. In its turn, the higher-energy *trans*-I conformer of 2FBA is unstable, decaying fastly into *cis*-I by tunneling (through a low barrier of only 13.9 kJ·mol^−1^; see Figure 4, bottom right panel) or into *trans*-II both via thermal over-the-barrier and tunneling (through a barrier of only 2.5 kJ·mol^−1^). The occurrence of these decay processes justify the experimental results described in [16], and are in agreement with the fact that this conformer was not observed in the microwave study (in any case, considering the relative energy of *trans*-I, even in the absence of the spontaneous tunneling decay reactions its population in the room temperature gas phase could be expected to be negligible).

In the case of 2CBA, the highest energy *trans*-I conformer could only be detected [17] upon deuteration of the carboxylic group, which reduces its tunneling decay rate substantially allowing its experimental detection under cryogenic conditions. The barrier for the *trans*-I→*cis*-I conversion is, for 2CBA, of 27.5 kJ·mol^−1^, which is still low enough to allow fast tunneling. On the other hand, the 2CBA intramolecularly hydrogen bonded conformer *trans*-II could be experimentally detected after its in situ photoproduction in a cryogenic matrix [17], but found to decay slowly by quantum mechanical tunneling into *cis*-II (half-live of ~10–30 min, at 9–45 K in a Xe matrix). The *trans*-II→*cis*-II energy barrier in 2CBA is 36.6 kJ·mol^−1^, i.e., intermediate between that of *trans*-II in 2FBA, which is a stable species (barrier: 40.4 kJ·mol^−1^) and the barriers for all other *trans*→*cis* isomerizations in BA, 2FBA and 2CBA (between 27.5 and 13.9 kJ·mol^−1^) that lead to fast tunneling and unstable *trans* conformers.

Taking into account the discussion above for 2FBA and 2CBA, the reasons why the *trans* conformer of benzoic acid was not experimentally detected both in the gas phase experiments [52,53,56] and in the matrix isolation studies [54,55] also became clear: the barrier for its conversion into the *cis* form is only 23.4 kJ·mol^−1^, intermediate between those for conversion of *trans*-I into *cis*-I forms in 2FCB and 2CBA, and lying within the range of values, which enables fast tunneling; the *trans* conformer of BA is then also unstable, even at cryogenic temperatures.

The conformational spaces of the three investigated di-substituted compounds, **2,6-difluoro-, 2-6-dichloro-, and 2-chloro-6-fluoro- benzoic acids** (abbreviated here as 26DFBA, 26DCBA and 2C6FBA, respectively; see also Figure 2) are relatively more simple than those of the mono-substituted compounds. In practical terms, all these compounds have only one *cis* and one *trans* conformer, though in the case of 2C6FBA three non-equivalent minima exist on the potential energy surface (one *cis* and two *trans*). No structural experimental data has been reported for the isolated molecules of these compounds.

The two conformers of 26DCBA have the carboxylic moiety exactly perpendicular to the plane of the ring, in order to minimize the repulsion between the chlorine *ortho* substituents and the oxygen atoms of the carboxylic group. Each form is two-fold degenerate, the *trans* conformer being higher in energy than the *cis* by 17.05 kJ·mol^−1^. On the contrary, for 26DFBA the conformers are 4-fold degenerate and the planes of the carboxylic group and the aromatic ring make angles of 47.1 and 26.6°, respectively for the *cis* and *trans* conformer. The *trans* conformer is 14.87 kJ·mol^−1^ higher in energy than the *cis* form. No intramolecularly hydrogen bonded conformers exist for these two molecules, as the required geometries would also imply strong repulsions between the ring halogen atoms and the oxygen atoms of the carboxylic moiety.

**Figure 6:**
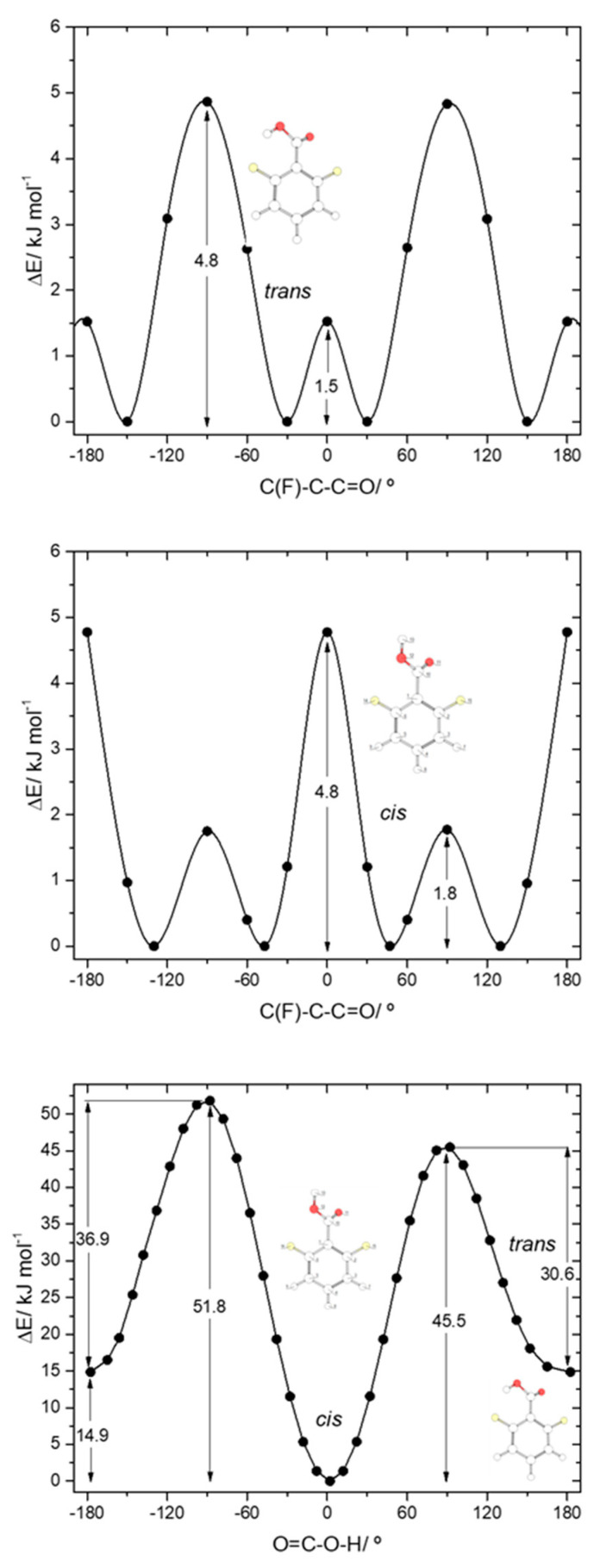
B3LYP/6-311++G(d,p) calculated potential energy profiles for internal rotation about the exocyclic C-C (for *cis* and *trans* arrangement of the carboxylic group) (*top and middle panels*) and about the C-O bond (connecting the *cis* and *trans* conformers; *bottom panel*) for 2,6-difluorobenzoic acid.

**Figure 7:**
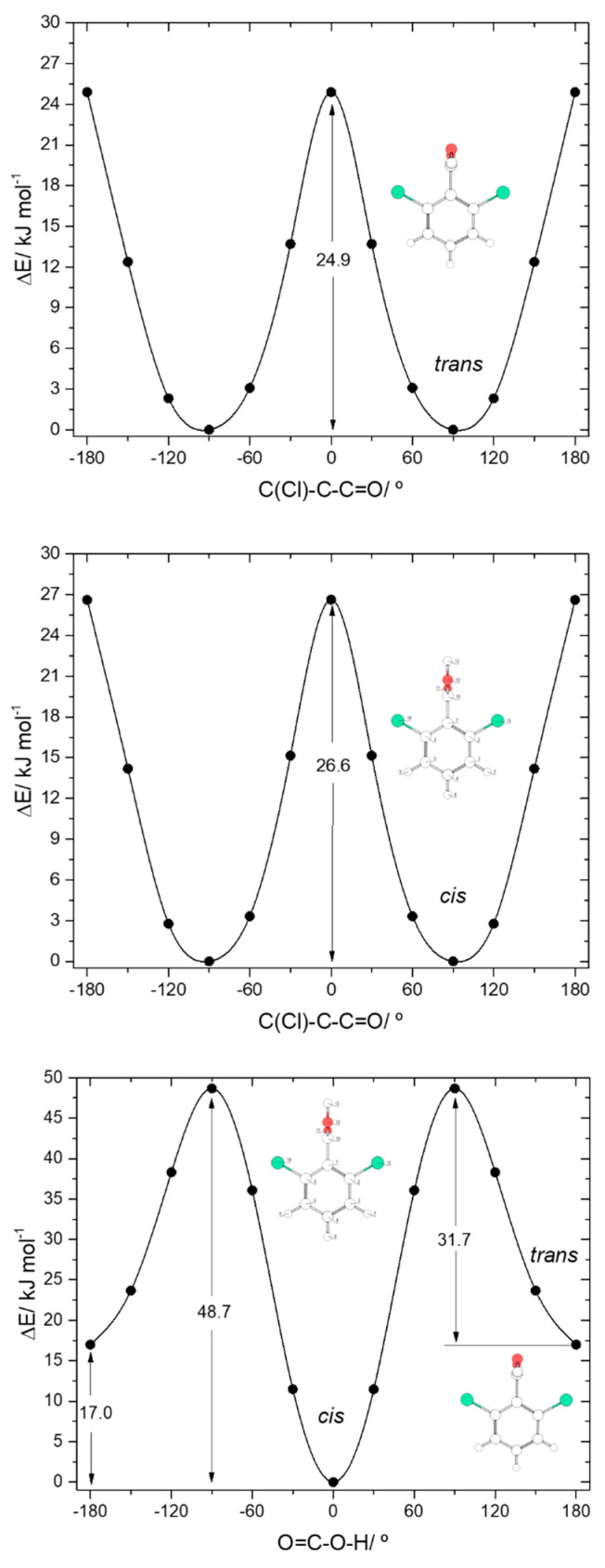
B3LYP/6-311++G(d,p) calculated potential energy profiles for internal rotation about the exocyclic C-C (for *cis* and *trans* arrangement of the carboxylic group) (*top and middle panels*) and about the C-O bond (connecting the *cis* and *trans* conformers; *bottom panel*) for 2,6-dichlorobenzoic acid.

The potential energy profiles for internal rotations about the C-O and exocylic C-C bonds in these two molecules are shown in Figure 6 and Figure 7.

The *trans*→*cis* energy barrier in 26DCBA is predicted as being 31.7 kJ·mol^−1^, which is intermediate between those associated with the *trans*-I→*cis*-I and *trans*-II→*cis*-II transformations in 2CBA (27.5 and 36.6 kJ·mol^−1^, respectively; see above). This result suggests that *trans* 26DCBA is unstable, decaying spontaneously to the *cis* form via tunelling if produced in some way. The barrier for the reverse *cis*→*trans* transformation (48.7 kJ·mol^−1^) is identical to those predicted for BA, 2FBA and 2CBA. The barriers of internal rotation about the C-C bond in 26DCBA are similar for both *cis* and *trans* carboxylic acid group conformations (26.6 and 24.9 kJ·mol^−1^, for *cis* and *trans* respectively), stressing the dominance of the Cl^…^O repulsive interactions at the planar transition states. These barriers are also similar to the overall energy barrier for internal rotation about the C-C bond in *trans* 2DCBA (25.5 kJ·mol^−1^; see Figure 5, top-right panel), which is also dominated by the repulsive Cl^…^O (and OH^…^H) interactions, but more than 3-times higher that in *cis* 2DCBA (7.8 kJ·mol^−1^; Figure 5, top-left panel), where the Cl^…^O repulsive interaction at the transition state is partially compensated by a stabilizing C-H^…^O= interaction.

Due to the asymmetrical position of the minimum energy conformations along the C-C internal rotation in both *cis* and *trans* 26DFBA relatively to the two fluoro- *ortho* substituents, the *trans*↔*cis* interconversion in this molecule can take place through two different transition states, depending on the direction of the rotation of the moving carboxylic hydrogen atom along the transformation (see Figure 6, bottom panel). The two barriers are, nevertheless, not much different, amounting to 30.6 and 36.9 kJ·mol^−1^ in the *trans*→*cis* direction and to 45.5 and 51.8 kJ·mol^−1^ in the reverse direction. The latter barriers are similar to *cis*→*trans* barriers found for the remaining studied molecules (including 2C6FBA; see below), allowing us to conclude that a barrier in the 45–52 kJ·mol^−1^ range is characteristics for the carboxylic group *cis*→*trans* transformation in this type of molecules. In the reverse (*trans*→*cis*) direction, the barriers found for 26DFBA are similar to those predicted for both 26DCBA and 2CBA (see above), being of intermediate size within the set of values for these barriers in the whole set of studied molecules, but they are significantly different from those observed for the *trans*-I→*cis*-I and *trans*-II→*cis*-II transformations in 2FBA (13.9 and 40.4 kJ·mol^−1^, respectively). As for the *trans* conformer of 26DCBA, the *trans* 26DFBA conformer shall be unstable and convert to the *cis* form spontaneously by tunneling.

The potential energy profiles for internal rotation about the exocyclic C-C bond in 26DFBA, for *cis* and *trans* arrangements of the carboxylic group (Figure 6, top and middle panels), reveal the 4 symmetry-equivalent minima in both cases, which can be grouped in two pairs, separated by a transition state where the carboxylic group and the aromatic ring are nearly perpendicular. Within each group, the two forms are connected by a planar transition state. For the *cis* arrangement of the carboxylic group, the first transition state is the lowest energy one (1.8 kJ·mol^−1^), while the planar transition state separating the members of each pair of symmerey-equivalent forms has an energy of 4.8 kJ·mol^−1^. This result highlights the dominance of the F^…^O repulsions at the planar *cis* transition state. On the other hand, for the *trans* arrangement of the carboxylic group the planar transition state has a lower energy (1.5 kJ·mol^−1^) than the perpendicular one (4.8 kJ·mol^−1^), a result that indicates that the O-H^…^F interaction established with one of the F *ortho* atoms partially compensates the effect of the repulsive interaction between the carbonyl oxygen atom and the second *ortho* fluoro-substituent. Compared with the C-C barriers in 26DCBA, the barriers in 26DFBA are much lower, mostly because of the much smaller size of the fluorine atom compared with the chlorine atom, which reduce the strength of the repulsive halogen-oxygen interactions in the fluorinated compound.

**Figure 8:**
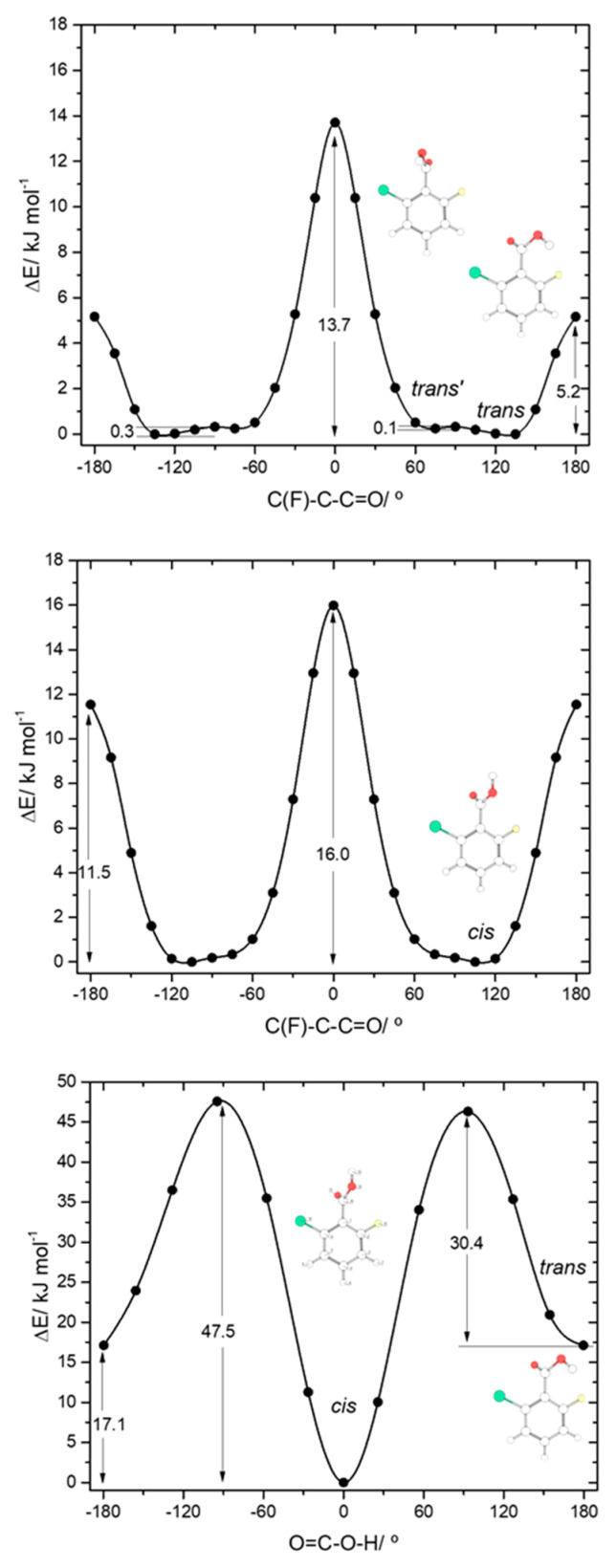
B3LYP/6-311++G(d,p) calculated potential energy profiles for internal rotation about the exocyclic C-C (for *cis* and *trans* arrangement of the carboxylic group) (*top and middle panels*) and about the C-O bond (connecting the *cis* and *trans* conformers; *bottom panel*) for 2-chloro-6-fluorobenzoic acid.

The asymmetrically substituted 2-chloro-6-fluorobenzoic acid has one *cis* conformer, which, as for the remaining molecules studied, is the lowest energy conformer, and is lower in energy than the *trans* conformer by 17.07 kJ·mol^−1^. As already mentioned, the *cis*→*trans* barrier (47.5 kJ·mol^−1^; see Figure 8) is similar to those in the other compounds, while the barrier for the reverse transformation amounts to 30.4 kJ·mol^−1^, similar to those found for 26DFBA, 26DCBA and 2CBA, and thus, as for these molecules, the *trans* conformer of 2C6FBA can also be expected to decay spontaneously by tunneling to the *cis* conformer. 2C6FBA has been investigated in a Xe matrix before [58] and, in consonance with these results, only the *cis* conformer was observed in the matrix. In the present study, we attempted to produce the *trans* conformer by using both UV and IR irradiation of the *cis* 2C6FBA conformer isolated in a Xe matrix (see Section 3.4), but we were unable to detect this form (as expected).

The *cis* and *trans* potential energy profiles for internal rotation about the exocyclic C-C bond in 2C6FBA are similar (see Figure 8, top and middle panels), with shallow wells at the position of the conformers. In the case of the *cis* conformer, the two symmetry-equivalent forms are separated by barriers of 16.0 and 11.5 kJ·mol^−1^, for the planar transitions states with the carbonyl oxygen atom positioned close to the fluorine and chlorine atoms respectively (and the acid oxygen atom positioned close to the second halogen atom). This result indicates that the sum of the energies associated with the F^…^O= and Cl^…^OH repulsions is larger than the sum of the Cl^…^O= and F^…^OH interactions, as expected taking into account the conclusions from the discussion made above for the mono-substituted compounds. In the case of the *trans* arrangement of the carboxylic group, rotation about the C-C bond reveals two non-equivalent 2-fold degenerated minima (named as *trans* and *trans’* in Figure 2 and also in Figure 8, top panel). However, the barrier separating *trans’* from *trans* (0.1 kJ·mol^−1^) stays below the zero-point energy for the τC-C torsional vibration of *trans’* (0.2 kJ·mol^−1^) so that this structure is better described as a vibrationally excited state of *trans*, resulting that 2C6FBA has a single 2-fold degenerate by symmetry *trans* conformer with experimental significance. The barriers separating the two symmetry equivalent *trans* forms of 2C6FBA amount to 13.7 and 5.2 kJ·mol^−1^, for the planar transitions states with the OH carboxylic group pointing to the chlorine and fluorine atoms, respectively. This result can be directly correlated with the relative strengths of the stabilizing O-H^…^F and O-H^…^Cl stabilizing interactions.

### 3.2. Other Molecular Properties: Internal Strain, Acidity, and Dipole Moment

As detailed in the previous section, the nature of the *ortho* substituents (H, F, Cl), and the relative amount of internal strain they introduce in the studied molecules, play an important role in determining their conformational preferences. Internal strain is also of fundamental importance in determining structural features like bond lengths, and in particular, within the carboxylic moiety, and, together with the relative indutive and resonance abilities of the substituents, also in defining the acidity and the dipole moment of the studied compounds.

**Figure 9:**
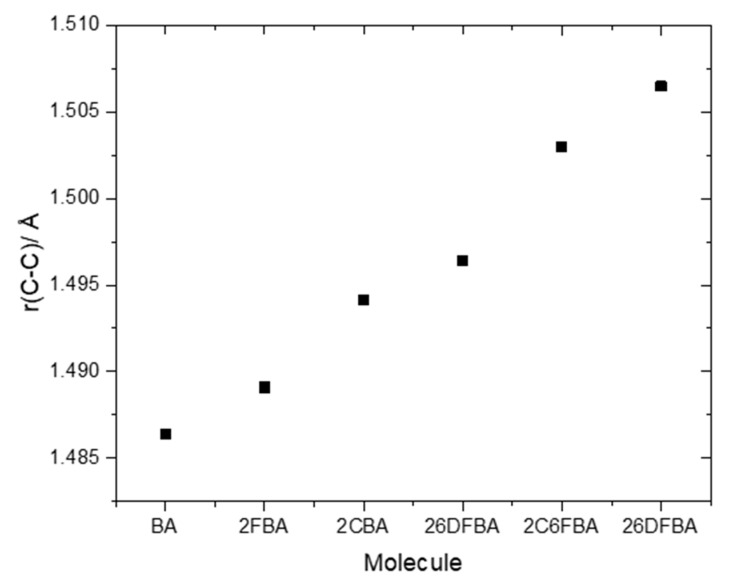
B3LYP/6-311++G(d,p) calculated bond lengths of the exocyclic C-C bond in the studied molecules.

The relative internal strain in the experimentally relevant *cis* conformers of the investigated molecules can be promptly estimated by considering the relative lengths of the exocyclic C-C bond connecting their carboxylic acid group to the aromatic ring. As seen in Figure 9, the C-C bond length increases in the order BA < 2FBA < 2CBA < 26DFBA < 2C6FCA < 26DCBA, as it could be anticipated (in the plot shown in Figure 9, the average of the C-C bond lengths in the two *cis* conformers for both 2FBA and 2CBA is used). As pointed in the previous section, from the practical point of view the *cis* conformers of benzoic acid and of the two mono-substituted compounds are planar, while those of the three di-substituted compounds are toughly non-planar. This fact influences decisively the properties of the carboxylic acid group, making the two sets of molecules intrinsically different regarding its characteristics.

**Scheme 1:**
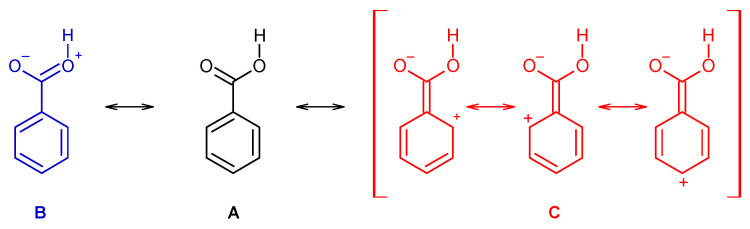
Mesomeric structures representing the resonance stabilization within the carboxylic group (**B**) and the competing cross conjugation with the aromatic ring (**C**). Structure **A** corresponds to the molecule canonical form.

Indeed, the resonance stabilization within the carboxylic group (represented in Scheme 1 by the structure **B**) is weak in benzoic acid and in the two mono-substituted compounds, due to the cross conjugation with the aromatic ring (**C**, in Scheme 1), which competes with the first effect. On the other hand, because of the non-planarity due to the stronger steric effects in the di-substituted derivatives, the cross conjugation is diminished in these compounds, so that the resonance stabilization within the carboxylic group becomes considerably more important. This has relevant structural implications, and it also plays a major role in determining the relative acidity of the two groups of molecules (for this latter property, one has also to count with the strain induced by the *ortho* substituents in the ionized carboxylate group of the conjugated bases of the acids, but for these species the effect can even be expected to be more relevant, due to the larger effective volume of the carboxylate moiety when compared with the non-ionized carboxylic acid group [49,50,51].

**Figure 10:**
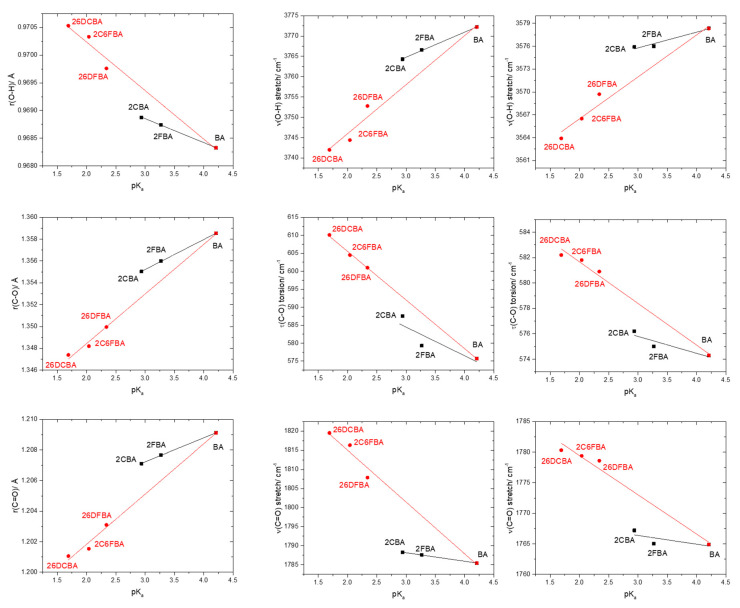
Correlation between the O-H, C-O and C=O bond lengths and of the ν(O-H), τ(C-O), and ν(C=O) vibrational frequencies of the experimentally relevant *cis* conformers and pK_a_ values (water; 25 °C) for the studied molecules. From *top-left* to *bottom-right* panels (descending by columns), the slopes, intercepts of the fitted lines and the R^2^ parameters of the adjustements are: (−4.318 × 10^−4^, 0.970, 0.99897; −8.755 × 10^−4^, 0.972, 0.97477), (2.750 × 10^−3^, 1.347, 0.99982; 4.550 × 10^−3^, 1.339, 0.99409), (1.560 × 10^−3^, 1.202, 0.99852; 3.290 × 10^−3^, 1.195, 0.99014), (6.162, 3746.3, 0.99771; 12.052, 3721.9, 0.96834), (−7.968, 608.4, 0.60329; −13.522, 632.6, 0.99916), (−2.217, 1794.8, 0.99791; −13.544, 1842.1, 0.97844), (2.063, 3569.6, 0.90715; 3.504, 3555.4, 0.96512), (−1.331, 579.8, 0.66553; −3.277, 588.2, 0.98402), (−1.436, 1770.7, 0.65948; −6.422, 1792.3, 0.96951). For both 2-fluorobenzoic acid and 2-chlorobenzoic acid, calculated bond lengths and vibrational frequencies are the means of the values obtained for the two *cis* conformers.

Figure 10 shows plots of the calculated C-O, C=O and O-H bond lengths, as well as of vibrational frequencies associated of these bonds in the studied molecules [the localized ν(O-H) and ν(C=O) stretching and the τ(C-O) torsion modes; ν(C-O) is extensively coupled with the δ(C-O-H) in-plane bending], as a function of their acidity, as expressed by the corresponding pK_a_ values in water at room temperature (25°) [25,26,27,28,29,59]. To fully understand the plots, one shall recall that both F and Cl have negative (electron withdrawing) indutive effect (–I), which is more important for fluorine than for chlorine (in view of the corresponding relative electronegativities), and positive (electron donating) mesomeric effect (+M), which is also stronger in fluorine compared to chlorine (due to the better orbital interactions of the 2*p* C or O orbitals with the closest in energy 2*p* orbitals of the fluorine atom, compared to the chlorine 3*p* orbitals). Noteworthy, when both +M and –I effects are operating simultaneously, both fluorine and chlorine are globally electron attractors (i.e., the –I effect dominates), but the much stronger +M effect of fluorine compared to chlorine reduces its total electron attractor power substantially compared to the later, so that fluorine becomes a total weaker electron attractor than chlorine. One consequence of this is that 2-fluorobenzoic acid has a lower acidity compared to 2-chlorobenzoic acid (the experimental pK_a_ values for 2FBA and 2CBA at 25 °C in water are respectively 3.27 and 2.94 [25,27]). Of course, both compounds are considerably more acidic than the unsubstituted benzoic acid (pK_a_ = 4.20 [59]).

The pK_a_ values for the difluoro- and dichloro- *ortho* substituted benzoic acids are 2.34 and 1.69, respectively [26,28], with 2-chloro-6-fluorobenzoic acid having an intermediate pK_a_ (2.04 [29]). In these compounds, besides the indutive and mesomeric effects of the substituents, the internal strain leading to non-planarity between the carboxylic group and the aromatic ring has also to be taken into account. The last, as shown in Figure 9, follows the order 26DFBA < 2C6FBA < 26DCBA (which is also the order of the degree of non-planarity of the *cis* conformers of the three molecules, whose angles made by the planes of the carboxylic group and the aromatic ring are 47.1°, 67.6°, and 90.0°, respectively, see Figure 2).

It is clear from the data plotted in Figure 10 that for the two groups of molecules (BA, 2FBA and 2CBA, in one side, and 26DFBA, 2C6FBA and 26DCBA in the other) the correlations between the O-H, C-O and C=O bond lengths within the carboxylic group as well as between the characteristic vibrational frequencies associated to these moieties (ν(O-H), τ(C-O), and ν(C=O)) and the pK_a_s follow different linear trends. As expected, the trend lines for all the correlations have a larger slope for the di-substituted molecules, where the number of halogen substituents is higher and the internal strain is large enough to make the molecules non-planar, thereby, increasing the importance of the stabilizing resonance effect within the carboxylic acid group (which directly correlates with the lability of the acid hydrogen). Interestingly, with a single exception the slopes of the trend lines for the di-substituted compounds in the plots shown in Figure 10 are approximately twice those for the mono-substituted compounds.

An increase in the acidity of the compounds correlates with a longer O-H, as well as with a shorter C-O bond (as expressed by the resonance structure **B** in Scheme 1). Alongside, the ν(O-H) stretching frequency decreases (since the bond becomes weaker), while the frequency of the τ(C-O) torsion increases (because the C-O bond increases its double bond character, implying a higher force constant for the torsional vibration). The results show also that the C=O bond length decreases with the acidity and, concomitantly, ν(C=O) increases. Since the electronic charge in the carbonyl π-bond shall reduce (see Scheme 1), these results indicate that the carbonyl σ-bond has to become stronger. In fact, this is in consonance with previous investigations on the behavior of the π and σ electronic systems in the carbonyl moiety [50,60], which have shown that the changes in the strengths of the two components of the carbonyl double bond in general take place in opposite directions, i.e., a weakening of the π-bond leads to strengthen the σ-bond, and vice-versa. It shall also be noticed that the correlations between pK_a_ and the vibrational frequencies do also verify if instead of the calculated frequencies one considers the experimentally measured ones in the gas phase (Figure 10, right-side panels), although the complex band profiles and band overlappings, observed in the experimental spectra introduce, in some cases, some uncertainty in the experimental frequencies.

**Figure 11:**
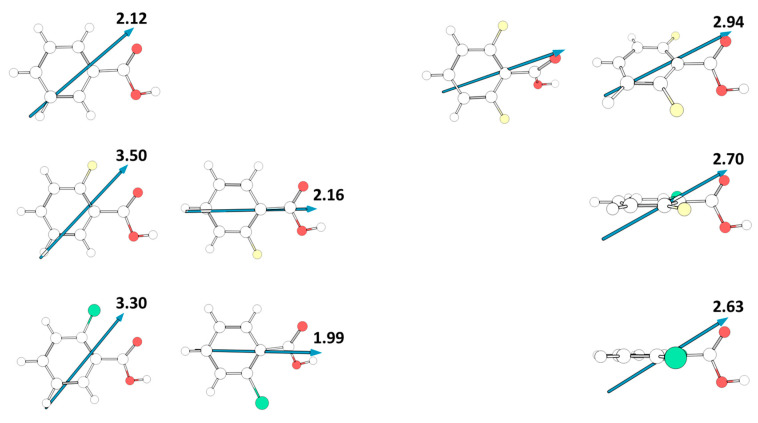
Calculated dipole moments (in Debyes; 1 Debye = ≈ 3.33564 × 10^−30^ C·m) for the studied molecules. For 2,6-difluorobenzoic acid, two representations are provided, one with the aromatic ring in the plane, and the other with the carboxylic acid substituent in the plane. In the case of the mono-substituted compounds (2FBA, 2CBA) the two *cis* conformers are shown.

Contrary to what happens in the case of acidity, the relative polarity of the different studied molecules, as measured by their total dipole moments, is mostly determined by the intrinsic polarity of the substituents, despite the dipole moment orientation reflects also their degree of planarity. Figure 11 shows the absolute value and the direction of the dipole moments for the experimentally relevant *cis* conformers of the various studied compounds. For benzoic acid and the mono-substituted compounds, the dipole moment is located in the molecular plane. In BA and in the *cis*-I conformers of 2FBA and 2CBA, it has approximately the same orientation, pointing from the C-H *meta* bond opposed to the carbonyl bond towards between this bond and the nearly located C-X (X = H, F, Cl) *ortho* bond, and increases along the series BA > 2CBA < 2FBA (2.12, 3.30, 3.50 D), while in the *cis*-II conformers of 2FBA and 2CBA the dipole moment points from the *para* C-H boind to the middle of the O=C-O angle of the carboxylic acid group, being also larger in the fluoro-substituted compound than in the chloro-substituted one (2.16 vs. 1.99 D). On the other hand, in the di-substituted compounds the dipole moment stays in the plane of the carboxylic acid and makes an angle of approximately 25° with the plane of the aromatic ring. In both 2F6CBA and 26DCBA the dipole moment points from below the *para* C-H bond up towards the carbonyl oxygen atom, while in the difluoro-substituted molecule it points from below the middle of the ring C-C bond connecting the *para* carbon atom to the *meta* one opposing to the carbonyl atom up towards this latter atom (see Figure 11). In the di-substituted molecules the order of increasing dipole moment is 26DCBA < 2C6FBA < 26DFBA, the magnitude of the dipole moments (2.63, 2.70, 2.94 D, respectively) being in between those of the *cis*-I and of the *cis*-II conformers of the mono-substituted molecules. If we consider the average values of the dipole moments of the two *cis* conformers in these latter molecules (2.83 and 2.64 D, respectively for 2FBA and 2CBA), the absolute values of the calculated dipole moments in the whole series of investigated substituted benzoic acids stay in the rather narrow range of values 2.63–2.94 D.

Unfortunately, to the best of our knowledge no gas phase experimental data on dipole moments for the studied molecules have been reported. For benzoic acid, values in the range 1.7–2.1 D have been reported in dioxane and CCl_4_ solution [61], and for 2FBA a value of 2.46 D has been reported [62], but without specification of the experimental conditions. These values seem to indicate that the dipole moments obtained in our calculations are probably within 10% accuracy, but a definitive conclusion on this matter must await for experimental confirmation.

### 3.3. Gas Phase IR Spectra

All the molecules studied are asymmetric tops, with 39 fundamental vibrations, all active in infrared. The experimentally relevant *cis* conformers of benzoic acid, 2FBA and 26DBA are *C*_s_ symmetry. The symmetry plane for the first two molecules corresponds to the molecular plane and the vibrations spawn the irreducible representations 27A′ + 12A′′, while in 26DCBA the plane of symmetry is perpendicular to the aromatic ring and contains the carboxylic group, the vibrations spawning the irreducible representations 23A′ + 16A′′. The *cis* conformers of 26DFBA and 2C6FBA, as well as the most stable conformer of 2CBA (*cis*-I), are *C*_1_ symmetry. On the other hand, as discussed in Section 3.1, from the practical point of view the *cis*-II conformer of 2CBA is planar, thus belonging to the symmetry point group *C*_s_ and, as in 2FBA, its vibrations spawn the irreducible representations 27A′ + 12A′′.

In the gas phase, infrared spectra are complicated by rotational contributions to the vibrational bands, which, together with the symmetry of the vibration, determine the band contours. For asymmetric top molecules of the size of those studied here, a detailed analysis of the band contours is a cumbersome task unless the molecule is approximately a symmetric top (either prolate, or oblate). In this case, band contour analysis may help to perform the assignment of bands that are not extensively overlapped or complicated by other effects (e.g., Fermi resonances and isotopic contributions, the latter being particularly relevant for the chloro-substituted molecules, since chlorine has two isotopes of large natural abundance).

The extent to which an asymmetric top molecule resembles a symmetric top is usually quantified by means of the Ray’s parameter *κ*, which can be estimated from the rotational constants, A, B, C (which directly relate with the moments of inertia, *I*_A_, *I*_B_, *I*_C_):

(2) $$\kappa =\frac{2B-A-C}{A-C}$$

*κ* = −1 for a prolate top (A > B = C), and *κ* = 1 for an oblate top (A = B > C).

**Figure 12:**
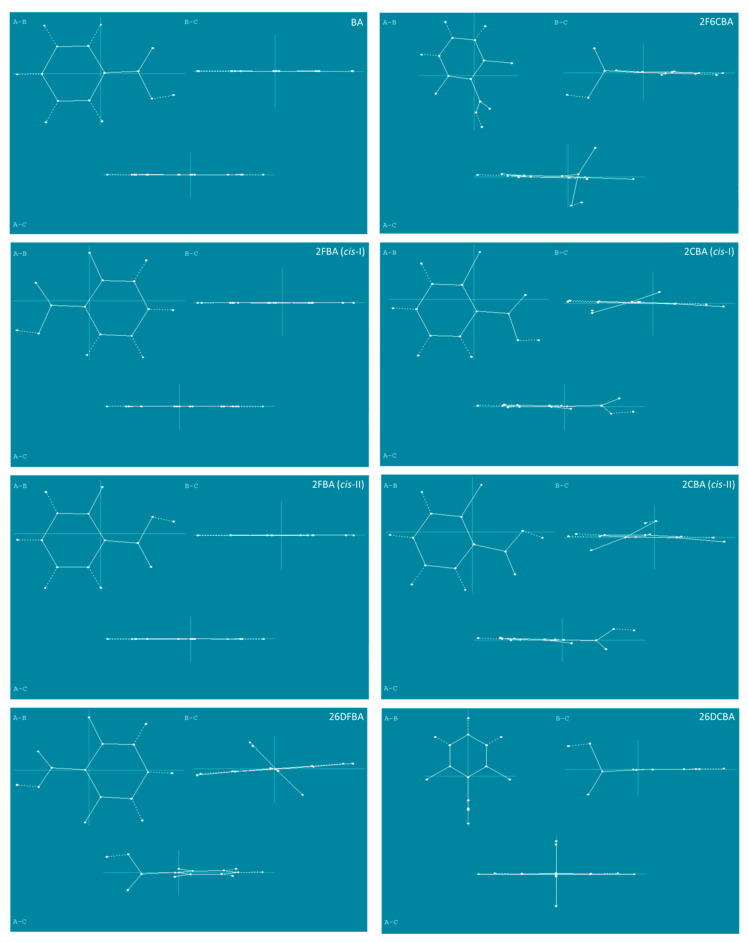
Projection of the experimentally relevant *cis* conformers of the studied molecules in the planes defined by the principal inertial axes, which are also represented in the figure, with lengths proportional to their associated rotational constants, A, B, C.

Table 1 shows the calculated rotational constants and the value of the *κ* parameter for the relevant conformers of the studied molecules. As it can be seen, only benzoic acid approaches the condition of being an approximate symmetric top (prolate; with *κ* = −0.80), with all the other molecules having |*κ*| < 0.50 with the exception of 2C6FBA, where *κ* = 0.73 (i.e., 2C6FBA has still a relatively significant oblate character). Considering these results, in the following discussion of the gas phase infrared spectra of the compounds, band contour analysis is only attempted for benzoic acid. In order to do this, it is relevant to known that the orientation of the principal inertial axes, which are depicted in Figure 12 for all the studied molecules.

For benzoic acid and the mono- and difluoro- substituted compounds, the A and B axes are nearly in the plane of the aromatic ring, the A axis being almost coincident with the *para* C-H bond and bisecting the O=C-O angle. In the case of benzoic acid, this means that all in-plane vibrations (*A*′ symmetry) shall show A, B or mixed AB band contours, while all out-of-the-plane vibrations (*A*′′ symmetry) shall exhibit C contour profiles. The orientation of the principal inertial axes in the remaining molecules can be seen in Figure 12, the striking case being 26DCBA, where the A axis (of lower momentum of inertia) stays in the plane of the aromatic ring, but aligned perpendicularly to the plane of symmetry of the molecule and passing through the two *ortho* chlorine atoms. In this regard, this molecule substantially differs from all the others being studied, which have the A axis located approximately in the plane of the aromatic ring and roughly oriented (or almost precisely oriented, as in benzoic acid) in a direction defined by the *para* C-H bond and the exocyclic C-C bond connecting the carboxylic acid substitent to the aromatic ring.

**Figure 13:**
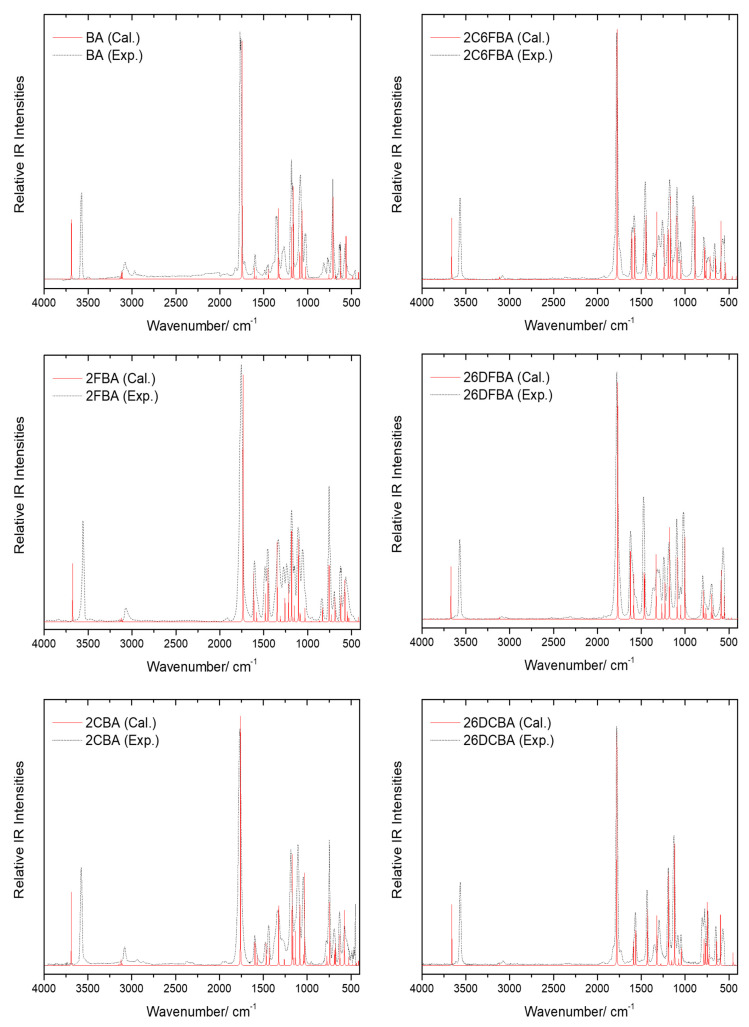
Gas phase infrared spectra of the studied compounds (black dashed traces) and B3LYP/6-311++G(d,p) calculated infrared spectra for their experimentally relevant conformers (red traces), in the 4000–450 cm^−1^ region. Calculated frequencies are scaled by 0.978. For simplicity, only the calculated spectra for the most stable conformers of 2FBA (*cis*-II) and 2CBA (*cis*-I) are shown in the figure (see also text).

The gas phase infrared spectra for all the six studied compounds are shown in Figure 13, together with the B3LYP/6-311++G(d,p) calculated spectra. For simplicity, only the calculated spectra of the most stable conformers of 2FBA (*cis*-II) and 2CBA (*cis*-I) are shown in the figure, since the spectra of the second populated conformers of these molecules in the gas phase (*cis*-I for 2FBA and *cis*-II for 2CBA) are very much similar to those of the corresponding dominant conformers [16,17].

The agreement between the calculated and experimental data is excellent, allowing the assignment of the spectra to be carried out straighforwardely. In Table 2, Table 3, Table 4, Table 5, Table 6 and Table 7, which show the proposed assignments, previously reported data obtained for jet-cooled gaseous benzoic acid [56] and for the monomer of this compound as well as of 2FBA, 2CBA and 2C6FBA isolated in low-temperature noble gas (Ar or Xe) matrices [16,17,54,55,58] are also presented. As decribed below, the present assignments agree with the general ones previously proposed for those molecules, but expand them providing assignment of additional bands and more evidence justifying the undertaken assignments (in particular for the unsubstituted compound). No vibrational studies have been reported on both 26DFBA and 26DCBA monomers hitherto.

According to the performed calculations, **benzoic acid** has 33 vibrations with frequencies lying in the studied spectral region (4000–450 cm^−1^), from which 26 are assigned in the present study. The 7 non-assigned vibrations have very low intensity (≤ 0.6 km·mol^−1^; see Table 2) and have never been observed experimentally with exception of the δ(CH) 0+–+0 mode (for the adopted notation of the vibrational modes see footnote *b* of Table 2) that has been tentatively assigned to a band at 1099 cm^−1^ in the IR spectrum of jet-cooled benzoic acid [56] (1110/1100 cm^−1^ in the Ar matrix spectrum of the compound [54,55]). In the gas phase spectrum of BA investigated in the present study (where the band profiles are, as expected, considerably broader than those recorded for the jet-cooled or matrix-isolated compound), the band ascribable to this vibration is hidden by the more intense band due to an essentially delocalized vibration with a significant contribution of the ν(C-O) stretching coordinate (1095 cm^−1^; predicted at 1090 cm^−1^ with an intensity of 44.6 km·mol^−1^). In the work on jet-cooled benzoic acid [56] only 19 modes of the molecule were assigned; moreover, the description of the modes was given in a very general way. In the matrix isolation investigations [54,55], the number of modes assigned within the 4000–450 cm^−1^ region is equal to the one achieved in the present work (plus the above mentioned δ(CH) 0+–+0 mode), and the observed frequencies in the two studies are within the expected agreement. However, the assignments done in the matrix isolation studies were in large amount based on low-level AM1 semi-empirical calculated spectral data. Therefore, the present study strongly improves the description of the infrared spectrum of gaseous benzoic acid.

The spectrum of BA is dominated by 6 intense bands which are ascribable to vibrations whose major contributors are associated with the polar carboxylic group, ν(O-H) (3578 cm^−1^), (C=O) (1765 cm^−1^), ν(C-O) (1354, 1078 cm^−1^) and δ(COH) (1354, 1184/1177 cm^−1^), as it could be anticipated. The band at 714 cm^−1^ is assigned to the γ(CH) all-in-phase out-of-the-plane bending mode (γ(CH) +++++, according to the notation adopted in the present study), having also a smaller contribution from the γ(C=O) coordinate. The calculated frequencies and relative intensities for these modes agree very well with the experimentally observed values (see Figure 13 and Table 2). The remaining two vibrations belonging to the carboxylic acid group which are expected to absorb in the studied spectral region give also rise to bands of considerable intensity, at 631 (δ(OCO)) and 574 (τ(C-O)) cm^−1^. The aromatic ring modes are also well-described by the calculations both, in terms of frequencies and intensities (see Figure 13 and Table 2), so that their assignment could also be made in a straightforward manner.

There are a few bands in the experimental spectra shown in Figure 13 that deserve here an additional comment. The first is observed at 1388 cm^−1^, and is probably due to the 2 × τ(ring)_1_ overtone enhanced by Fermi interaction with the fundamental mode assigned to the mixed {ν(C-O), δ(COH)} mode. The second is observed at 560 cm^−1^ and can be tentatively attributed to the combination tone τ(ring)_3_ + w(COOH) (calculated value: 404 + 155 = 559 cm^−1^). The last band is observed at 817 cm^−1^, and shall be ascribed to an unknown impurity, since it is absent in the spectra of both the jet-cooled and matrix-isolated monomer of benzoic acid and it is also not present in the spectrum of gaseous benzoic acid available in the Wiley Spectral Database [64].

**Figure 14:**
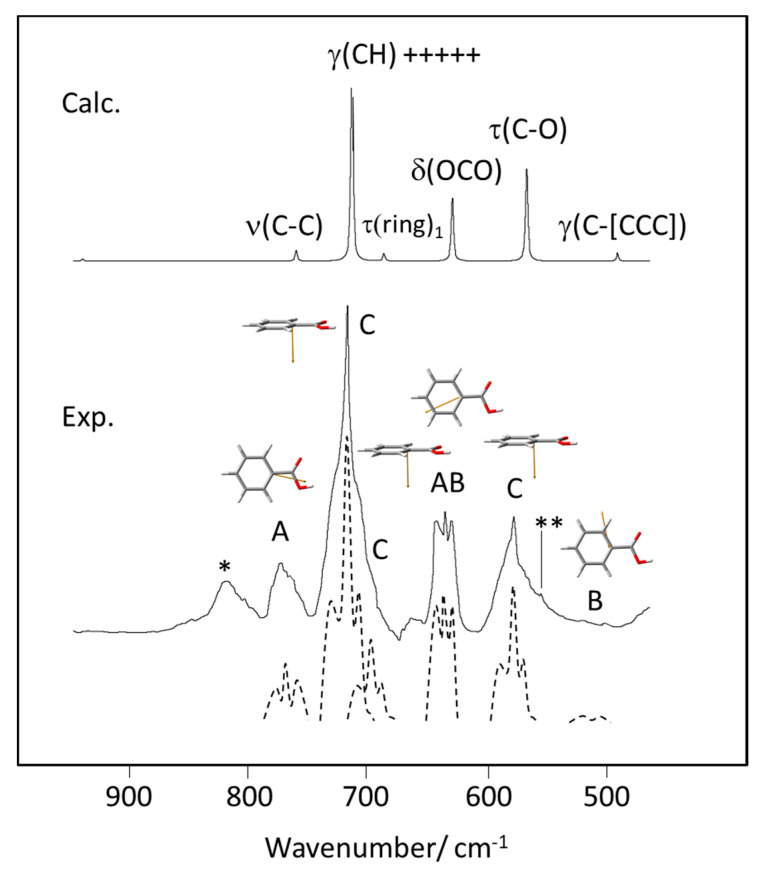
900–450 cm^−1^ spectral region of the experimental (Exp.) gas phase infrared spectrum of benzoic acid, with indication of the band contour type (A, B, AB, C). The dashed figures are approximate deconvolutions of the experimental bands, drawn with increased resolution to highlight the characteristic bands profiles. The calculated (Calc.) spectrum is shown at the top of the figure, with indication of the band assignments. The drawings of the molecule show the the transition dipole moments (in orange color) for the different vibrations (see Figure 12 for orientation of the principal axes of inertia of the molecule). *-band due to an unknown impurity; **-band due to the combination tone τ(ring)_3_ + w(COOH) (calculated values: 404 + 155 = 559 cm^−1^ (see Table 2).

The band contour analysis proved to be particularly interesting in the 800–450 cm^−1^ range, where bands predicted to exhibit different profiles (A, AB, B and C) are observed. As shown in Figure 14, the profiles of the experimental bands fit nicely the predicted contour types, giving additional support to the proposed assignments.

In the case of **2-fluorobenzoic acid**, from the 32 vibrations predicted by the calculations to absorb in the investigated spectral region (4000–450 cm^−1^), 27 were assigned to bands observed in the experimental spectra (see Table 3). Three of the 5 modes not assigned have very low predicted IR intensities (less than ~1 km·mol^−1^), while the remaining two are expected to give rise to bands of small intensity at about the positions of the very intense bands of the {ν(C-O), δ(COH)} (1340 cm^−1^) and {ν(C-O), δ(CH) 0++–} (1111/1105 cm^−1^) modes, and are then buried underneath those bands.

Like for benzoic acid, the infrared spectrum of 2FBA is dominated by intense bands originated in the carboxylic acid substituent, e.g., ν(O-H) (3576 cm^−1^), ν(C=O) (1765 cm^−1^), ν(C-O) (1340, 1184, 1111/1105 cm^−1^), δ(COH) (1340, 1184 cm^−1^), and τ(C-O) (575 cm^−1^). Also, as with BA, the γ(CH) all-in-phase out-of-the-plane bending mode (γ(CH) ++++) in 2FBA gives rise to an intense band at 755 cm^−1^. All these bands are well predicted by the calculations (see Figure 13 and Table 3).

The ν(CF) stretching mode is assigned to the band at 1238 cm^−1^, in fairly good agreement with the predicted value (1217 cm^−1^). As suggested in [16], the in-plane (δ(CF)) and out-of-the-plane (γ(CF)) bending modes give some contribution to the vibrations predicted at 531 cm^−1^ (observed at 525 cm^−1^) and 516 cm^−1^ (with a very low predicted intensity and not observed experimentally), respectively, which are, however, better described as skeletal ring deformation modes, due to major contributions from the ring coordinates. The δ(CF) and γ(CF) modes have been reassigned to the calculated vibrations with frequencies at 344, and 244 cm^−1^, respectively, rectifying the approximate description given previously for these modes as ring skeletal deformations [16].

For 2FBA, besides the lowest energy *cis*-II conformer, the higher energy *cis*-I conformer shall also contribute to the spectrum. As mentioned before, the infrared spectra of the two conformers are predicted to be very similar and in the gas phase spectrum the evidence of *cis*-I can only be clearly noticed in a few spectral regions, where this conformer gives rise to intense bands at positions sufficiently distinct from the corresponding bands of the dominant *cis*-II conformer. The bands of *cis*-I predicted to have a large intensity and obeying the condition of not being to close in frequency to those of *cis*-II are calculated at 3691 (ν(O-H)), 1760 (ν(C=O)), 1226 (ν(CF)), 1175 ({ν(C-O), δ(COH)}, 1048 (δ(ring)_1_), 841 (δ(ring)_2_) and 557 (τ(C-O)) cm^−1^. In the case of the ν(O-H) and τ(C-O) modes, no clear bands due to *cis*-I could be identified, though the bands assigned to these modes are considerably asymmetric, with a more extended wing in the side where the bands due to *cis*-I are expected (respectively for higher and lower frequencies). In all the other cases, the bands due to the *cis*-I conformer are clearly seen in the experimental spectrum, at frequencies matching well the predicted values (1778, 1245, 1162, 1059 and 845 cm^−1^; see also Figure 13 and Table 3).

The presence of two conformers at equilibrium in the gas phase occurs also for **2-chlorobenzoic acid**. Being nearly degenerate, the expected relative population of the two conformers is determined by their symmetry. As mentioned in Section 3.1, these considerations lead to an expected population of the slightly more stable *cis*-I conformer of 2CBA that is approximately twice that of the less stable *cis*-II conformer. However, compared to 2FBA, the spectra of the two *cis* conformers of 2CBA are considerably more similar, so that in this case the band overlapping is more extensive. In fact, only one band in the experimental gas phase IR spectrum of 2CBA could be clearly assigned to the *cis*-II conformer. This band is observed at 1371 cm^−1^, and is ascribable to the {ν(C-O), δ(COH)} mode, in fairly good agreement with the calculated frequency (1344 cm^−1^).

As for BA and 2FBA, the most intense bands in the infrared spectrum of 2CBA are due to vibrations mostly localized in the carboxylic acid fragment, ν(O-H) (3576 cm^−1^), ν(C=O) (1767 cm^−1^), ν(C-O) (1334, 1103, 1083 cm^−1^), δ(COH) (1334, 1185 cm^−1^) and τ(C-O) (576 cm^−1^), plus the γ(CH) all-in-phase out-of-the-plane bending mode (γ(CH) ++++) (746 cm^−1^). The stretching of the *ortho* C-Cl bond contributes significantly to two bands, being strongly mixed with the ν(C-O) coordinate in the vibration giving rise to the intense band at 1103 cm^−1^ (predicted at 1083 cm^−1^), and with both the δ(OCO) and δ(ring)_3_ coordinates in the vibration giving rise to the band observed at 690 cm^−1^ (predicted at 677 cm^−1^). The in-plane and out-of-the-plane bending CCl vibrations are predicted to originate bands of low intensity outside the investigated spectral region (respectively at 293 and 205 cm^−1^; see Table 4).

As a whole, 25 from the 27 fundamental vibrations of 2CBA predicted to absorb above 450 cm^−1^ with an intensity larger than 2.5 km·mol^−1^ have been assigned in the present investigation, the two non-assigned modes corresponding to low-intensity vibrations whose bands are hiden by intense bands due to other closely located in frequency modes, specifically {ν(C-O), δ(COH)} and τ(C-O) (see Figure 13 and Table 4). In the matrix isolation study of 2CBA [17] only 14 modes were assigned.

The general spectroscopic pattern described above for BA, 2FBA and 2CBA in relation to the dominance of the bands originated in the carboxylic group regarding intensities is also observed for the studied **di-substituted compounds**. For both 26DFBA and 26DCBA no previous vibrational data have been reported. In here, 24 from 25 fundamental vibrations with intensities over 1 km·mol^−1^ predicted to occur in the studied spectral region (4000–550 cm^−1^) were assigned in the spectrum of 26DFBA, while for 26DCBA all 23 vibrations matching these criteria were ascribed to bands in the experimental spectrum (Table 5 and Table 7). The symmetric stretching vibration of the *ortho* fluorine substituents in 26DFBA is observed at 1295 cm^−1^ as a mid intensity band (predicted at 1266 cm^−1^), while the anti-symmetric mode gives rise to an intense band at 1020 cm^−1^ (predicted at 1002 cm^−1^). As for the mono-fluoro-substituted compound, the CF stretching frequencies in 26DFBA are underestimated by ca. 20 cm^−1^ by the calculations upon scaling. Interestingly, the unscaled calculated frequencies almost exactly match the experimental ones in both molecules (1294, 1024 versus 1295, 1020 cm^−1^ for 26DFBA, and 1244 vs. 1238 cm^−1^ for 2FBA), and the same trends are also observed for 2C6FBA (1266 cm^−1^, calculated unscaled versus 1258^−1^, observed versus 1239 cm^−1^, calculated scaled; see Table 6). The in-plane and out-of-the-plane CF_2_ bending vibrations in 26DFBA as well as the CCl_2_ bending modes in 26DCBA are predicted to absorb below 550 cm^−1^, i.e., outside the studied spectral region. All bands associated to these vibrations are predicted to have very low intensity (< 3 km·mol^−1^; see Table 5 and Table 7).

The infrared spectrum of 2F6CBA isolated in a cryogenic Xe matrix has been reported before [58] and assigned with help of calculated data obtained at the B3LYP/6-311++G(d,p) level of theory. The interpretation of the gas phase spectrum of the compound closely follows that made in the matrix isolation study, though a few bands are reassigned, in particular the streching of the exocyclic C-C bond, now assigned to the band at 779 cm^−1^ (which has also contributions from the δ(OCO), γ(C=O) and γ(CH) +++ coordinates; see Table 6). This band was previously mis-assigned to the stretching vibration of the CF bond, which is now reassigned to the band at 1258 cm^−1^ that appears at approximately the same frequency as the identical vibration in 2FBA (1238 cm^−1^). The νCCl stretching (not assigned in the matrix isolation study) is ascribed to the experimentally observed band at 1151 cm^−1^, in good agreement with the calculated data (1149 cm^−1^) and also not very much different from the frequency of the νCCl mode in 2CBA (1103 cm^−1^; calculated: 1083 cm^−1^; see Table 4). Both νCF and νCCl coordinates also contribute (in opposition of phase) to the intense band observed at 909 cm^−1^. Another way to describe the vibrations of the CF and CCl bonds is to state that the two stretchings combine anti-symmetrically to give rise to the band at 909 cm^−1^, while the putative symmetric combination is replaced by vibrations where each one of the coordinates combine with δ(CH) ring bending modes (see Table 6). According to the calculations, the in-plane bending associated with the CF and CCl bonds also combine to give rise to symmetric (predicted at 230 cm^−1^) and anti-symmetric vibrations (predicted at 418 cm^−1^). Interestingly, the out-of-plane deformations are predicted to be independent from each other. For 2C6FBA, all 23 vibrations predicted to absorb in the studied spectral region with an intensity >1 km·mol^−1^ are here assigned to experimental bands in the gas phase spectrum of the compound.

### 3.4. Photochemistry under Matrix Isolation Conditions

**Figure 15:**
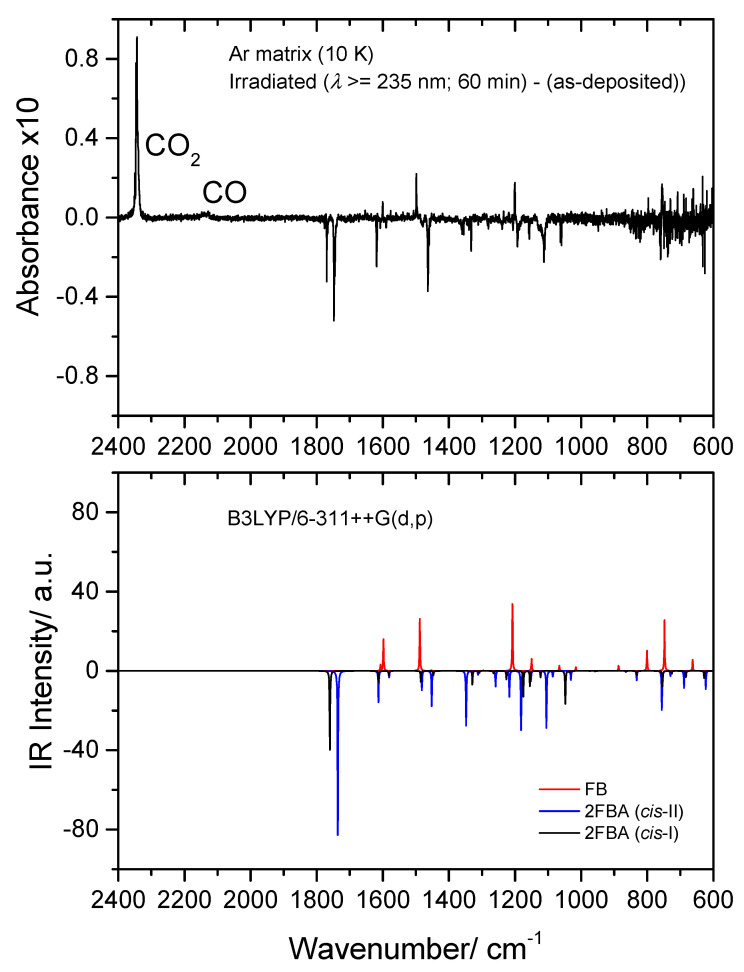
*Top*: 2400–600 cm^−1^ region of the difference IR spectrum: irradiated Ar matrix of 2-fluorobenzoic acid (2FBA) *minus* as-deposited matrix. *Bottom:* Simulated difference spectrum based on the B3LYP/6-311++G(d,p) calculated IR spectra of 2FBA (*cis*-I *and cis*-II conformers; bands pointing down) and the observed photoproducts: CO_2_, CO, and fluorobenzene (FB) (bands pointing up). The calculated wavenumbers were scaled by 0.978, and the calculated intensities were multiplied by arbitrary factors in order to fit better the observed spectrum (in a ratio of ~2:1:3, 2FBA *cis*-II:2FBA *cis*-II:FB).

**Figure 16:**
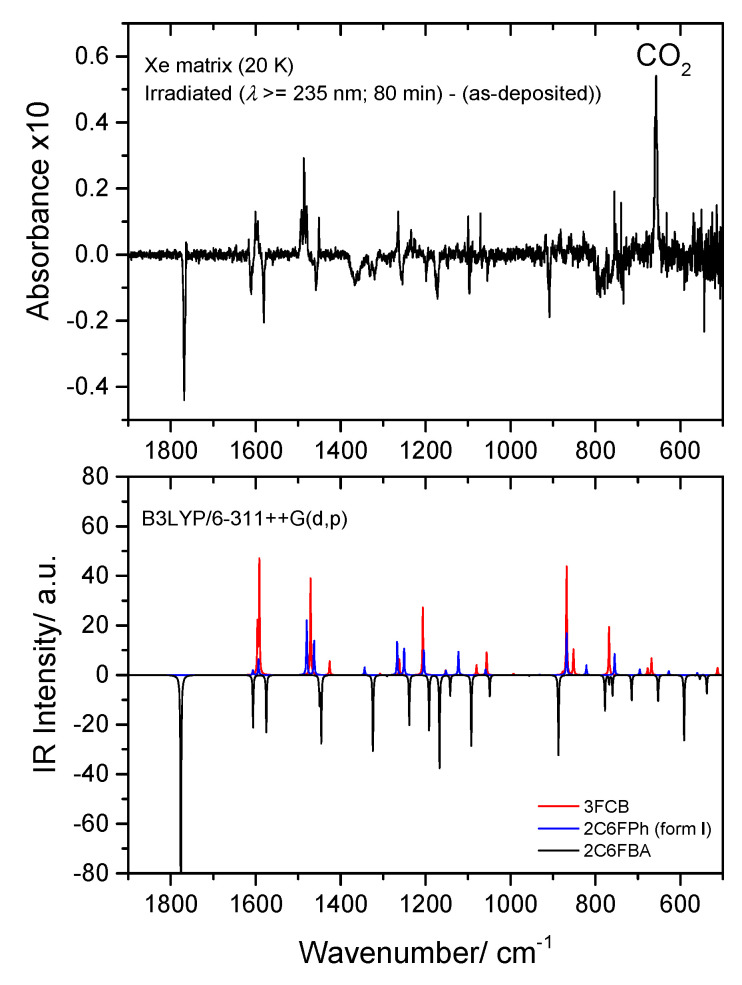
*Top*: Low frequency region of the difference IR spectrum: irradiated Xe matrix of 2-chloro-6-fluorobenzoic acid (2C6FBA) *minus* as-deposited matrix. *Bottom:* Simulated difference spectrum based on the B3LYP/6-311++G(d,p) calculated IR spectra of 2C6FBA (*cis* conformer; bands pointing down) and the observed photoproducts: CO_2_, 2-chloro-6-fluorophenol (2C6FPh) and 3-fluoro-chlorobenzene (3FCB) (bands pointing up). The calculated wavenumbers were scaled by 0.978, and the calculated intensities of the reactant and of the different photoproducts were multiplied by arbitrary factors in order to fit better the observed spectrum (in a ratio of ~3:2:1, 2C6FBA:3FCB:2C6FPh).

**Figure 17:**
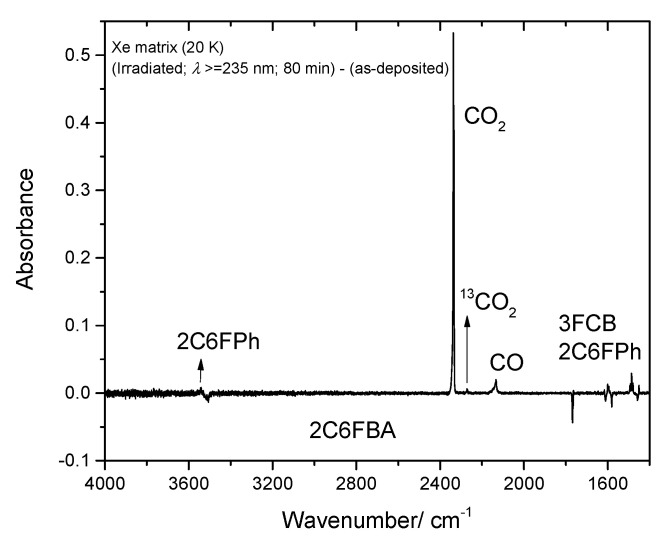
High frequency region of the difference IR spectrum: irradiated Xe matrix of 2-chloro-6-fluorobenzoic acid (2C6FBA) *minus* as-deposited matrix. Bands that arise due to the photoproduced CO_2_ (including its ^13^C isotopologue), CO, 2-chloro-6-fluorophenol (2C6FPh) and 3-fluoro-chlorobenzene (3FCB) are pointing up, while bands due to the reactant (*cis* conformer) are pointing down. The lower frequency region shown in this figure (<2000 cm^−1^) was included to allow a faster comparison of the intensities of the bands with those presented in Figure 16.

Two of the compounds were selected as targets for photochemical experiments, 2FBA and 2C6FBA. In these experiments, monomers of the compounds were isolated in cryogenic Ar (2FBA) or Xe (2C6FBA) matrices and subjected to narrowband UV (λ ≥ 235 nm) irradiation. The spectra of the as-deposited matrices are identical to those reported before [16,58]. Upon irradiation, new bands appeared in the infrared spectra, which are due to the formed photoproducts, while concomitantly the bands of the reactant decrease of intensity. Figure 15 summarizes the results obtained for 2FBA, and Figure 16 and Figure 17 those found for 2C6FBA.

For both compounds characteristic bands of CO_2_ and CO were observed in the spectra of the photolysed matrices, indicating that decarboxylation and decarbonylation reactions take place upon UV irradiation, decarboxylation being the major process. In the case of 2C6FBA, the decarboxylation:decarbonylation reaction branch is estimated to be ~2:1, based on the relative intensities of the bands due to the two photoproducts. For this compound, both photoproducts accompanying CO_2_ and CO, i.e., 3-fluoro-chlorobenzene and 2-chloro-6-fluorophenol (2C6FPh), respectively, were observed. The photoreactions in the case of 2FBA appear to be less effective, and the decarboxylation strongly dominates, precluding experimental detection of 2-fluorophenol, the product that is expected to be formed together with CO. It is also interesting to note that 2-chloro-6-fluorophenol, which has two conformers differing in orientation of the phenolic OH group, is produced in the lowest energy conformer I, where the OH group is intramolecularly H-bonded to the fluorine *ortho* substituent. Also, it is worth noticing that in the case of 2FBA the two conformers trapped in the cryogenic matrix (*cis*-I and *cis*-II) seem to react at nearly equal rates.

Both photodecarboxylation and photodecarbonylation are commonly observed reactions of carboxylic acids, and have been described for this type of compounds isolated in cryogenic matrices and subjected to UV irradiation [65,66]. General mechanistic insigths on these processes escape the scope of the present article and can be found in [65,66,67,68,69]. Nevertheless, one shall point out that the recombination of radical species produced in the excited states after UV irradiation, which have been described as the primary photoproducts of UV-photolysis of carboxylic acids as a result of the cleavage of the C-O or C-C bond alpha to the carbonyl, is particularly easy in the solid matrix media, due to the cage-confined nature of the processes. Under these conditions, stable species like CO_2_ and CO shall be promptly formed, as observed. In addition, quantum chemical calculations (performed at the CASSCF level of theory) on benzoic acid [69] have shown that the transition states for cleavage of those bonds have the carboxylic group and the aromatic ring perpendicular to each other. This may explain, at least partially, the observed larger efficiency of the photoreactions observed for 2C6FBA (whose molecules have these two groups almost perpendicular in the starting geometry) compared to 2FBA (where the two groups are in the same plane).

It shall also be noticed that a previous photochemical investigation of matrix-isolated 2CBA has been reported [17]. In that study, monomeric 2CBA isolated in a low-temperature (9 K) argon matrix was subjected to irradiation using a super-high-pressure mercury broad-band source assembled with a short-wavelength filter (λ > 240 nm). The authors were able to induce conversion of the initially deposited *cis* conformers into the high-energy *trans* conformers, which were then found to spontaneously convert back to the initial forms by quantum mechanical tunneling (as it has already been mentioned in this article; see Section 3.1). However, no information about simultaneous occurrence of any photolysis reaction has been provided.

### 3.5. Structural Characteristics of the Crystalline Materials

**Figure 18:**
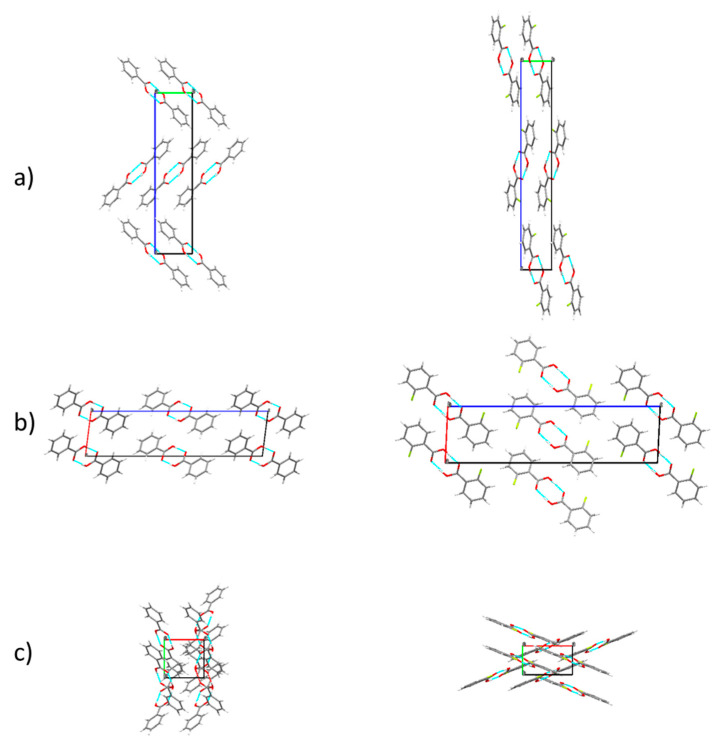
Crystal packing for BA (*left*) and 2FBA (*right*) as viewed from the (**a**) (*top*), (**b**) (*middle*) and (**c**) (*bottom*) crystallographic axes. Data from Refs. [20,30], respectively.

**Figure 19:**
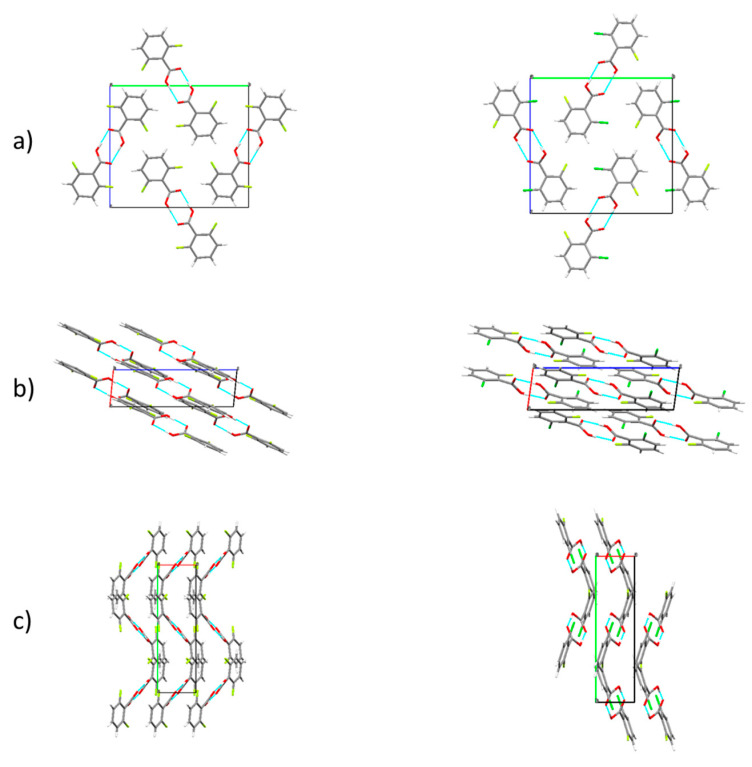
Crystal packing for 26DFBA (*left*) and 2C6FBA (*right*) as viewed from the (**a**) (*top*), (**b**) (*middle*) and (**c**) (*bottom*) crystallographic axes. Data from [22], and [24], respectively.

**Figure 20:**
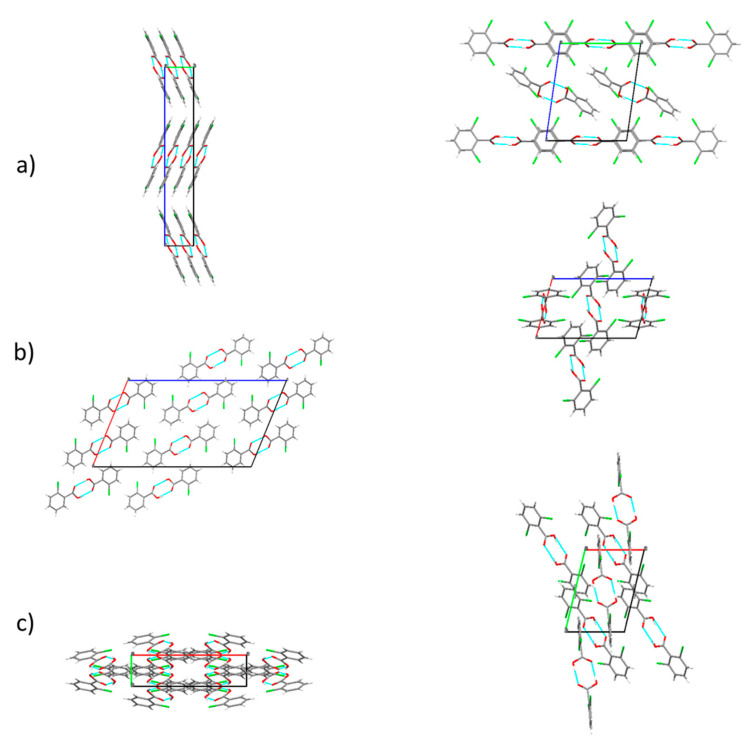
Crystal packing for 2CBA (*left*) and 26DCBA (*right*) as viewed from the (**a**) (*top*), (**b**) (*middle*) and (**c**) (*bottom*) crystallographic axes. Data from [21], and [23], respectively.

The crystal structures for all the compounds investigated here have been previously reported [20,21,22,23,24,30]. Table 8 summarizes the crystal data, while Figure 18, Figure 19 and Figure 20 depict the crystal packing schemes observed for the different compounds. All compounds except 26DCBA crystalyze in the monoclinic system, the crystals belonging to the P2_1_/c (or P2_1_/n) space group N° 14 (BA, and all fluoro-containing compounds) or to the closely related C2/c space group N° 15 (2CBA). The crystals of the dichloro-substituted compound are triclinic, space group P$\bar{1}$, and also differs in the number of non-equivalent molecules in the unit cell (*Z′*), which is 2, instead of 1 as for all other compounds. The fundamental dissimilarity of the crystal of 26DCBA compared to those of the remaining compounds can be ascribed to the different packing constrains imposed by the favored nearly perpendicular arrangement of the carboxylic acid substituent and the aromatic ring in the 26DCBA molecules (see Section 3.1). The average value of the angle between the planes of the carboxylic acid substituent and the aromatic ring (*φ*) in 26DCBA is ~85°, slightly smaller than in the isolated molecule (90.0°), but much larger than in the remaining studied compounds. For all compounds, the monomeric units exist in a carboxylic acid *cis* conformation and are associated in centrosymmetric dimers connected by two O-H^…^O intermolecular hydrogen bonds. In the BA crystal, the molecules are planar, as for the isolated molecule. The crystals of the two mono-substituted compounds (2FBA and 2CBA) show molecules slightly non-planar: for 2CBA, the angle between the carboxylic acid substituent and the aromatic ring is ~14°, very much similar to that predicted for the most stable conformer of the molecule, *cis*-I (18.0°; see Figure 1); for 2FBA, this angle is ~10°, while the minimum energy *cis* structures of the isolated molecule are planar. Interestingly, 2FBA is the only compound among all those studied here where the conformer selected to form the crystal (*cis*-I) is not the most stable conformer found for the isolated molecule (*cis*-II, as discussed in Section 3.1). Both 26DFBA and 2C6FBA crystals are constituted by non-planar molecules where the *φ* angle is somewhat smaller than in the corresponding minimum energy structures of the isolated molecules (~34° vs. 47.1° in the case of 26DFBA, and ~46° vs. 67.6°, for 2C6FBA). In practical terms, the trends described above lead to geometries adopted by the different *ortho* halogeno-susbtituted benzoic acids molecules in crystalline phase that are more similar to each other than for the isolated molecules, this being particularly noticeable for the compounds crystallyzing in the monoclinic system.

It is interesting to note the similarity of the cell parameters for the pairs BA/2FBA and 26DFBA/2C6FBA (see Table 8), which follows the general pattern of greater structural similarity between these pairs of molecules already pointed out in the previous sections of this article. In this regard, the two chloro-substituted compounds, 2CBA and 26DCBA, have unique characteristics, though in the case of the mono-substituted compound this is mostly due to the different number of molecules in the unit cell, which is twice those of the remaining compounds (indeed, the cell parameters for 2CBA can also be made in close correspondence with those of the pair 26DFBA and 2C6FBA if one takes ½ of the value of the *c* axis and interchanges the *a* and *b* axes; see Table 8).

The cell volumes for the crystals of the different compounds shown in Table 7 follow the expected order, tacking into account the size of the respective molecules, i.e., BA < 2FBA < 26DFBA < ½2CBA < 2C6FBA < 26DCBA, though the cell volume of the crystal of the unsubstituted compound (BA) given in Table 8 is comparatively larger. However, this is in a large amount due to the higher temperature used in the X-ray structure determination measurements, since neutron diffraction studies performed at 130 K [30] render a cell volume for BA crystal of only 589 Å^3^.

Intermolecular interactions in the studied crystals are here characterized in details using the Hirshfeld surface analysis approach [43,70,71] and the CE-B3LYP method [42,43].

Hirshfeld surfaces are obtained from electron distributions that are calculated as sums of spherical atomic electron densities [43,70,71]. The Hirshfeld surface of a molecule in a crystal defines the region where the electron distribution given by the sum of the electron densities of the spherical atoms of a given molecule (the *promolecule*) exceeds that from all other promolecules in the crystal. Structure-related properties can then be mapped on the Hirshfeld surface. The normalized contact distance (*d*_norm_) is calculated from the distances of a given point of the surface to the nearest atom outside (*d*_e_) and inside (*d*_i_) of the surface (normalized by the corresponding van der Waals radii, $r_{i}^{vdw}$ and $r_{e}^{\;vdw}$, respectively), as defined by Equation (3), and allows the identification of the regions of the molecule where intermolecular interactions are more important [71,72]. Additionally, the combination of *d*_e_ and *d*_i_ in the form of a two-dimensional (2D)-fingerprint plot allows to condense information about the intermolecular contacts present in the crystal [71,73,74,75]. The 2D-fingerprint plots provide a visual summary of the frequency of each combination of *d*_e_ and *d*_i_ across the surface of a molecule, thus indicating not just which intermolecular interactions are present, but also the relative area of the surface corresponding to each kind of interaction, which is a measure of the relative amount of each interaction in the crystal:

(3) $$d_{\mathrm{norm}}=\frac{d_{i}-r_{i}^{vdw}}{r_{i}^{vdw}}+\frac{d_{e}-r_{e}^{vdw}}{r_{e}^{vdw}}$$

The CE-B3LYP method has been described in details in refs. [42,43], and alows the partition of the interaction energies in a crystal in components which are of electrostatic, polarization, dispersion and exchange-repulsion nature. As described in Section 2 of this article, this method enables the computation of lattice energies (E_lat_), which can be then related with sublimation enthalpies ΔH_sub_(*T*).

**Figure 21:**
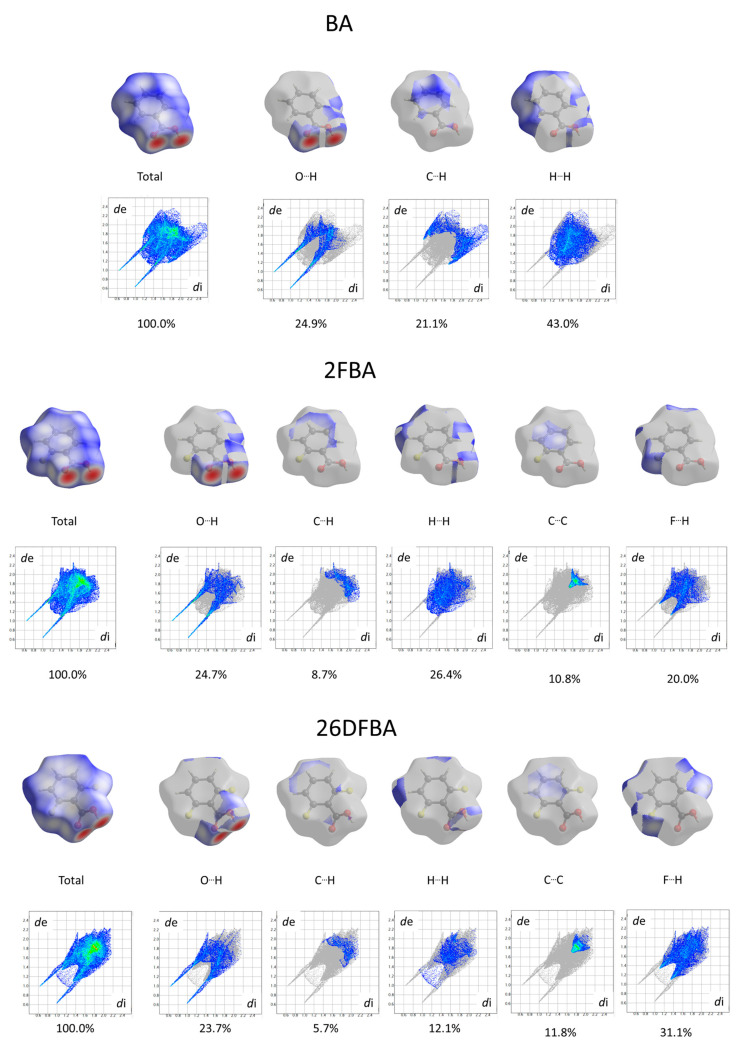
Total and atom^…^atom *d*_norm_ Hirshfeld plots and 2D-fingerprint plots (*d*_e_, *d*_i_) for BA, 2FBA and 26DFBA. Grey, colors means no interaction, blue-to-red colors mean increasing strength of interaction (in the *d*_norm_ Hirshfeld plots) or increased number of interactions (in the 2D fingerprint plots).

**Figure 22:**
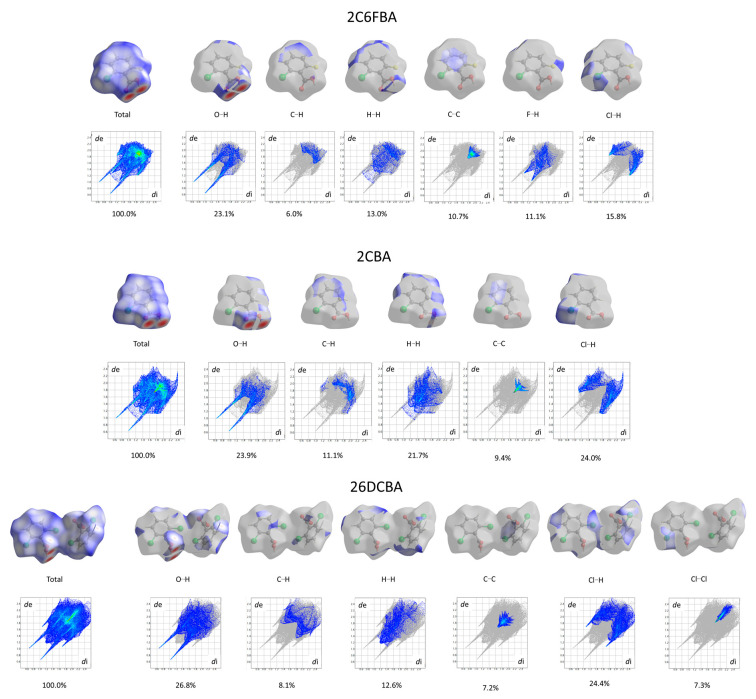
Total and atom^…^atom *d*_norm_ Hirshfeld plots and 2D-fingerprint plots (*d*_e_, *d*_i_) for 2C6FBA, 2CBA and 26DCBA. Grey, colors means no interaction, blue-to-red colors mean increasing strength of interaction (in the *d*_norm_ Hirshfeld plots) or increased number of interactions (in the 2D fingerprint plots).

**Figure 23:**
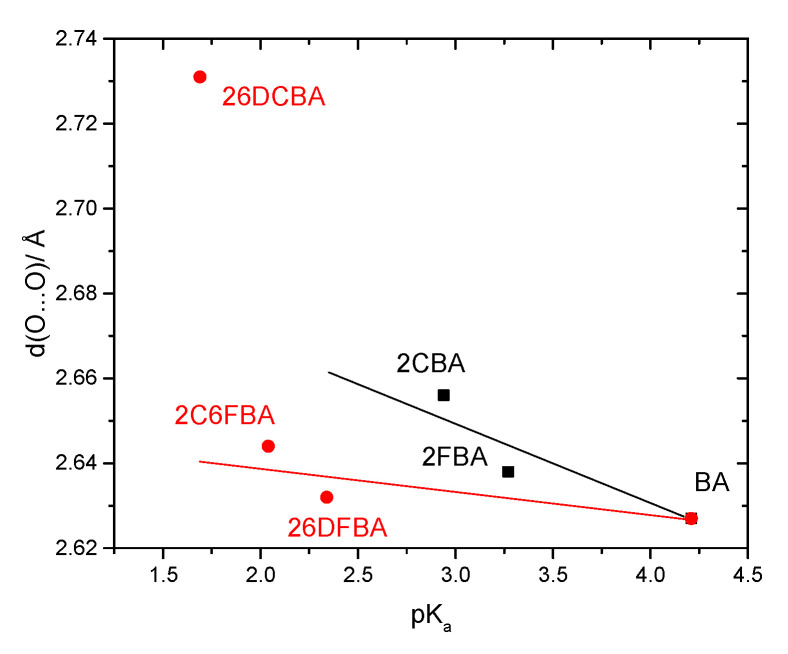
Intramolecular d(O^…^O) distances in the dimers existing in the crystals of the studied compounds [20,21,22,23,24,30] vs. pK_a_ values (water; 25 °C).

Table 9 summarizes the results of the Hirshfeld analysis performed on the crystals of the studied compounds regarding the relative importance of the different intermolecular interactions, expressed as atom^…^atom interactions according to the *d*_norm_ signatures. The total and atom^…^atom *d*_norm_ plots and 2D-fingerprint plots (*d*_e_, *d*_i_) are shown in Figure 21 and Figure 22. It can be seen that, as expected, the O^…^H interactions are of fundamental importance in all crystals, since they are mostly related with the intermolecular H-bond interactions joining individual molecules in the centrosymmetric dimers present in the crystals. The O^…^H interactions percent contribution is in the short range of 23.1–24.9% in the series of compounds studied, testifying the similar nature of this type of interaction in all of them. It is interesting to point out that the strengths of the intermolecular O-H^…^O interactions in the dimers present in the crystals, as measured by the O^…^O distances (the shorter the distance, the stronger the H-bonding) correlate approximately in an inverse way with the pK_a_ of the compounds (grouping the mono-substituted and di-substituted compounds; Figure 23), once again 26DCBA being a notorious exception to this trend.

Besides the O^…^H interactions, in the BA crystal the other two most relevant types of interactions are the H^…^H and C^…^H contacts, accounting for 43.0 and 21.1% of the Hirshfeld surface *d*_norm_ values, respectively. As shown in Figure 21, the H^…^H interactions are mapped on the Hirshfeld surface around all ring hydrogen atoms and are related with the dispersion interactions involving these atoms, which occur both paralelelly and perpendicularly to the aromatic ring plane, as seen in Figure 18. On the other hand, the C^…^H interactions are a measure of the C-H^…^π interactions involving the π system of the aromatic ring and the *meta* and *para* hydrogens of neighboring molecules, as shown in Figure 18. Note that in the crystal of BA the π-π stacking between the aromatic rings is limited, since the rings of neighbor dimers are dephased (see Figure 18), what justifies the small importance of the C^…^C interactions in the crystal (only 3.6%; Table 9). This strongly contrasts with what happens in the case of 2FBA, where the π-π stacking between adjacent rings is perfect and, consequently, the C^…^C interactions increase of importance (10.8%) at cost of the C^…^H interactions (which account only for 8.7% of the Hirshfeld *d*_norm_ surface area), and are mapped, as expected, above the aromatic ring carbon atoms (see Figure 18 and Figure 21). In the crystal of 2FBA, besides the O^…^H and C^…^C interactions, the most importante interactions are the H^…^H and H^…^F ones, accounting respectively for 26.4 and 20.0% of the Hirshfeld *d*_norm_ surface area and representing mostly H^…^H dispersive and C-H^…^F-C bond-polar interactions. Interestingly, the F^…^F interactions are practically inexistent (0.2%), in consonance with the fact that in the crystal the atoms close to the fluorine atoms are essentially hydrogen atoms.

A similar picture can be traced for the difluoro-substituted compound, whose percentual assignment of the Hirshfeld *d*_norm_ surface area to the diferent atom^…^atom interactions is very much identical to that of 2FBA (see Table 9 and also Figure 18, Figure 19 and Figure 21). The major difference is the increase of percentage allocated to the F^…^H interactions and the reduction of that assigned to the H^…^H interactions, as expecting considering the replacement of one H atom by a F atom in going from 2FBA to 26DFBA.

The patterns of atom^…^atom interactions in the crystals of 2CBA and 26DCBA generally follow those discussed above for the two fluoro-substituted compounds, as it can be noticed by the percentual values for the different interactions shown in Table 9, and also by comparing the plots depicted in Figure 21 and Figure 22 for these molecules. It shall, however, be emphasised that the specific geometry adopted by the molecules of 26DCBA, with the planes of the aromatic ring and of the carboxylic group nearly perpendicular to each other, leads to a reduction of the π-π staking between the aromatic rings (as measured by the C^…^C interaction, which has the lowest percent value among all these four molecules; see also Figure 20 for 26DCBA crystal packing), and also to an increase of the relevance of the Cl^…^O interactions (which are by far more important in 26DCBA than in 2CBA, and also much more important than the F^…^O interactions in both 2FBA and 16DFBA; see Table 9).

Finally, in the crystal of 2C6FBA, whose structural organization resembles that of 26DFBA (Figure 19), the intermolecular atom^…^atom interactions do also resemble those opperating in the difluoro-substituted compound, with the relevant O^…^H, H^…^H and C^…^C interactions having identical percentual contributions to the Hirshfeld *d*_norm_ surface area (Table 9 and Figure 21 and Figure 22). Naturally, the percentage assigned to the F^…^H interactions in 26DFBA (31.1%) are split in 2C6FBA among the F^…^H (11.1%), Cl^…^H (15.8%) and F^…^Cl (6.3%) interactions. Additionally, the Hirshfeld *d*_norm_ surface maps for 2C6FBA clearly reveal that the F^…^H interactions occur predominantly with the *para* hydrogen atom, while the Cl^…^H interactions involve essentially the *meta* hydrogen atoms (Figure 22), in consonance with its characteristic crystal packing, shown in Figure 19. For 26DFBA, the F^…^H interactions extend to both *para* and *meta* hydrogen atoms (Figure 19 and Figure 21).

**Figure 24:**
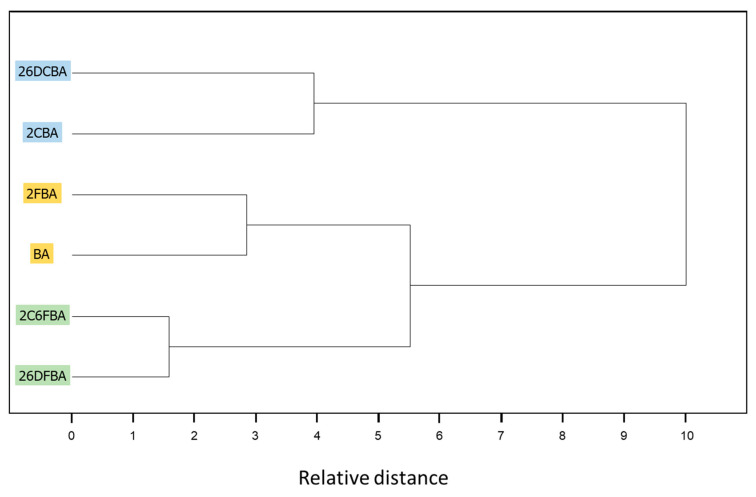
Results of clustering analysis (Ward’s method with squared Euclidian distance) of the CE-B3LYP data for the crystals of the studied compounds. For this analysis, the UnscramblerTM CAMO software (Version 10.5) [76] was used.

The results obtained using the CE-B3LYP interaction energy decomposition model [42,43] are summarized in Table 10. A comparative analysis of the data for the 6 studied crystals was done by using the Ward’s clustering method [77] with squared Euclidian distances, and is presented in Figure 24. The clustering analysis clearly shows the similarity of the pairs of crystals BA/2FBA and 26DFBA/2C6FBA already pointed out above, with the latter pair being that whose components show the highest degree of similarity. The crystals of the chlorosubstituted compounds (2CBA and 26DCBA) are also grouped together in the clustering analysis, but their degree of similarity is considerably smaller than those exhibited by the remaining pairs of crystals, as seen by the relative distances of the branching points of the dendogram for the different groups.

The electrostatic and polarization components of the intermolecular interactions (E_ele; E_pol) are larger for all fluoro-containing compounds (including 2C6FBA), compared to the chloro-substituted ones (see Table 10), as it could be expected taking into account the relative electronegativities of the two halogen atoms. The maximum value of these terms are observed for 26DFBA, and are ca. 30%, and ~70% higher, respectively, than for 26DCBA. Interestingly, the results indicate that these terms are also large for the unsubstituted BA.

The dispersion component of the intermolecular interactions (E_dis) is, as expected, smaller in BA, compared with all halogenated benzoic acids (with the notorious exception of 26DCBA), and in these latter, also as it could be anticipated that it is larger in the di-substituted compounds than in the mono-substituted ones. The fact that the crystal of 26DCBA appears (again) as an outlier shall be ascribed to its significant structural differences compared to the remaining crystals, which were already pointed out above.

In its turn, the exchange-repulsion component (E_rep) of BA has an intermediate value, being larger than those found for 2CBA and 26DCBA and smaller than those calculated for all the fluoro-containing derivatives. Assuming a similar compactness of the crystals, these results can be correlated with the relative hardness of the halogen atoms, fluorine being a harder atom than hydrogen and chlorine being considerably softer [78,79].

The CE-B3LYP calculated lattice energies of the crystals E_lat_ = E_tot (see Table 10) appear in the following order: 26DFBA < 2C6FBA = 2FBA ≈ BA < 2CBA < 26DCBA. From the obtained values of E_lat_, the sublimation enthalpies were estimated as described in Section 2 as being 109 (26DFBA), 102 (2C6FBA and 2FBA), 101 (BA), 94 (2CBA) and 82 (26DCBA) kJ·mol^−1^. The estimated values are expected to have an absolute error within 8 kJ·mol^−1^ [42], so that the obtained results compare fairly well with the available experimental data: 94.4 ± 0.8, 93.3 ± 1.2 and 79 ± 2 kJ·mol^−1^, for 2FBA, BA and 2CBA, respectively [80,81,82] (no experimental data is available for the remaining compounds).

We have also looked to the influence of the crystal environment on the properties of the aromatic ring. Aromaticity can be estimated using the harmonic oscillator model of aromaticity (HOMA) index [83,84,85], which is defined as,

(4) $$\mathrm{HOMA}=1-\frac{257.7}{6}\sum_{i=1}^{6}{\left(1.388-r_{i}\right)}^{2}$$

where *r_i_* are the C-C bond lengths.

Table 11 presents the values of the ring C-C bond lengths for the isolated molecule, as calculated in the present study, and those obtained experimentally for the crystals [20,21,22,23,24,30]. For both situations, the less aromatic rings are present in the mono-substituted compounds due to pronouncedly different nature of the *ortho* substituents, with 2CBA being the compound exhibiting the smaller HOMA index. Interestingly, the aromaticity index in the di-substituted compounds assumes higher values than in BA, both in the crystalline state and for the isolated molecules. It is also worth noticing that, with no exceptions, the intermolecular interactions present in the crystals lead to a reduction of the aromaticity degree of the aromatic moiety (from a reduction of ~0.15% in the HOMA index, in both 26DFBA and 26DCBA, to ~5% in 2CBA).

## 4. Conclusions

In this article, the structure and properties of the *ortho* mono- and di-substituted fluoro- and chloro- benzoic acids were investigated in a comparative basis. First, the potential energy landscape of the isolated molecules of the compounds were characterized using DFT calculations performed with the B3LYP functional and the 6-311++G(d,p) basis set. Special attention was given to the influence of the interactions between the carboxylic group and the *ortho* halogen substituents as well as the nature of these later on the structures and properties of the studied series of compounds. The structures of the relevant conformers of the molecules were rationalized and used to interpret the experimental data obtained for the isolated molecules of the compounds, and in particular, their vibrational spectra in the gas phase and isolated in low-temperature inert matrices, and the photochemistry of selected illustrative compounds in the latter experimental conditions. The stability of the higher-energy *trans* carboxylic conformers regarding their spontaneous conversion by quantum mechanical tunneling to the most stable *cis* forms was evaluated. The conclusions derived from the analysis of the potential energy landscapes of the molecules regarding this matter were found to duly explain previously reported experimental data [16,17,58] and were used to make predictions regarding the stability of the *trans* conformers of the molecules not yet experimentally investigated (BA, 26DFBA and 26DCBA). The structures of the crystals reported previously in the literature [20,21,22,23,24,30] were revisited and discussed, also in a comparative basis. Intermolecular interactions in the different crystals and their effects on the properties of the compounds were evaluated through Hirshfeld surface analysis, the CE-B3LYP energy decomposition model and HOMA index. The structural characteristics of the crystals were correlated with thermodynamic data. On the whole, this article presents a detailed comprehensive investigation of the *ortho* fluoro- and chloro- substituted benzoic acids both as isolated molecules and in the crystalline phase.

## Figures and Tables

**Table 1:** Calculated rotational constants (GHz) and Ray’s parameter for the experimentally relevant conformers of the studied molecules.

Molecule	A	B	C	*κ*
BA	3.8809001	1.2246207	0.9308807	−0.80
2FBA (*cis*-II)	2.2877050	1.2087660	0.7908917	−0.44
2FBA (*cis*-I)	2.2641140	1.2114724	0.7891939	−0.43
26DFBA	1.5243775	1.1493339	0.6953489	0.10
2CBA (*cis*-II)	1.4673930	1.1808936	0.6676423	0.28
2CBA (*cis*-I)	1.4469943	1.1976874	0.6621492	0.36
2C6FBA	1.1257278	1.0541388	0.5898361	0.73
26DCBA	1.0938018	0.7895163	0.4960684	−0.02

**Table 2:** Experimental (Exp.) and B3LYP/6-311++G(d,p) calculated (Calc.) infrared spectra of monomeric benzoic acid, and band assignments *^a^*.

Exp.	Exp. [56]	Exp. [54,55]	Calc.		Approximate Description *^b^*	Sym- metry	Contour Type *^c^*
Gas Phase	Cooled-jet	Ar Matrix, 12 K	B3LYP				
ν	ν	ν	ν	I^IR^			
3578	n.i.	3567	3689	99.4	ν(O–H)	*A′*	A
3124	n.i.	3098	3138	2.5	ν(CH) 0000+	*A′*	AB
3104	n.i.	3079	3132	4.6	ν(CH) +0000	*A′*	AB
3083	n.i.	3068	3118	12.7	ν(CH) 0+++0	*A′*	A
3060	n.i.	3041	3109	10.7	ν(CH) 0+0–0	*A′*	B
3031	n.i.	3012	3097	0.4	ν(CH) 0+–+0	*A′*	A
1765	1752	1755/1752/1751 /1743	1746	395.8	ν(C=O)	*A′*	AB
1599	1609	1606	1606	19.2	ν(CC) +–++–+	*A′*	A
1585	1591	1590	1586	5.3	ν(CC) +0–+0–	*A′*	AB
1481	n.obs.	1496/1493	1489	1.8	ν(CC) +0– –0+, δ(CH) ++0– –	*A′*	B
1451	1455	1456	1448	15.7	ν(CC) 0+00–0, δ(CH) 0+++0	*A′*	AB
1388/1354 *^d^*	1387	1383/1380 *^d^*	1333	117.2	ν(C–O), δ(COH)	*A′*	AB
1328	1347	1347	1321	8.1	ν(CC) +–+–+–, δ(CH) +++++	*A′*	AB
1284/1269 *^e^*	1278/1255*^e^*	1278/1275/1271/ /1255/1251/1248 *^e^*	1310	2.1	ν(CC) +0+–0–	*A′*	A
1184	1187	1185	1184	86.7	δ(COH), δ(CH) 0+0-0	*A′*	AB
1177	1173	1169	1163	153.8	δ(COH), δ(CH) +–0–+	*A′*	AB
n.obs.	1099	1110/1100	1158	0.6	δ(CH) 0+–+0	*A′*	AB
1095	1084	1086	1090	44.6	ν(C–O), ν(CC) – –0++0, δ(CH) 0000+	*A′*	AB
1078	1063	1066/1063	1065	114.6	ν(C–O), ν(CC) 0000+0, δ(CH) +0000	*A′*	AB
1026	1026	1027	1023	20.7	δ(ring) _1_	*A′*	AB
n.obs.	n.obs.	n.obs.	995	0.4	δ(ring) _2_	*A′*	A
n.obs.	n.obs.	n.obs.	987	0.1	γ(CH) +–+–+	*A″*	C
n.obs.	n.obs.	n.obs.	976	0.1	γ(CH) +–0+–	*A″*	C
947	n.obs.	937	939	1.7	γ(CH) +0–0+	*A″*	C
n.obs.	n.obs.	n.obs.	845	0.1	γ(CH) ++0– –	*A″*	C
n.obs.	n.obs.	n.obs.	806	0.1	γ(C=O)	*A″*	C
770	767	767/759	758	8.1	ν(C–C)	*A′*	A
714	710	713/711	711	136.8	γ(CH) +++++, γ(C=O)	*A″*	C
688	688	687	684	5.8	τ(ring)_1_	*A″*	C
631	631	628	626	48.9	δ(OCO)	*A′*	AB
n.obs.	587	568	618	0.2	δ(ring)_3_	*A′*	AB
574	571	565	563	71.5	τ(C–O)	*A″*	C
504	n.i.	491	486	6.1	γ(C-[CCC])	*A′*	B
n.i.	n.i.	421	423	12.1	τ(ring) _2_	*A″*	C
n.i.	n.i.	n.obs.	404	0.5	τ(ring) _3_	*A″*	C
n.i.	n.i.	n.i.	375	4.8	δ(ring) _4_	*A′*	AB
n.i.	n.i.	n.i.	212	1.5	γ(COOH)	*A′*	AB
n.i.	n.i.	n.i.	155	0.8	w(COOH)	*A″*	C
n.i.	n.i.	n.i.	64	0.9	τ(C–C)	*A″*	C

*^a^* Wavenumbers (ν) in cm^−1^, calculated infrared intensities (I ^IR^) in km·mol^−1^; n.obs., not observed; n.i., not investigated. *^b^* Approximate description was obtained using the vibrations animation module of Chemcraft [63]; ν, stretching; δ, bending; w, wagging; γ, rocking; τ, torsion; the symbols +, – and 0 placed in front of the ν(CC), ν(CH), δ(CH) and γ(CH) ring coordinates indicate the movement (distending, compressing, staying, respectively) of the related bonds, starting from that located *ortho* to the C=O bond of the carboxylic group and proceeding anti-clockwise through the ring. *^c^* Band contours in gas phase spectra predicted according to the calculated transition dipole moment vector orientation. *^d^* Fermi interaction with 2 × τ(ring)_1_. *^e^* Fermi interaction with 2 × δ(OCO).

**Table 3:** Experimental (Exp.) and B3LYP/6-311++G(d,p) calculated (Calc.; *cis*-II conformer) infrared spectra of monomeric 2-fluorobenzoic acid, and band assignments *^a^*.

Exp.	Exp. (This Work; Also [16])	Cal.		Approximate Description *^b^*	Sym- metry
Gas Phase	Ar Matrix, 10 K	B3LYP			
ν	ν	ν	I ^IR^		
3576	3558/3555	3676	96.4	ν(O–H)	*Aτ*
3100	3069	3137	4.9	ν(CH) ++++	*A′*
n.obs	n.obs.	3132	1.1	ν(CH) +0– –	*A′*
3087	3026	3121	6.7	ν(CH) +– –+	*A′*
3060	n.obs.	3107	2.8	ν(CH) 0+–+	*A′*
1765/(1778) *^c^*	1748/1747/1745/1744	1736	407.6	ν(C=O)	*A′*
1611	1619/1618/1617	1613	79.9	ν(CC) +–++–+	*A′*
1582	1593/1591/1590/1588	1581	16.0	ν(CC) +0–+0–	*A′*
1490	1506/1504/1493/1489/1487	1482	46.6	ν(CC) 00+00–, δ(CH) ++– –	*A′*
1458	1464/1463/1461	1452	88.5	ν(CC) 00+00–, δ(CH) 0++0	*A′*
1340	1395/1390/1388/1361/1360	1348	131.3	ν(C–O), δ(COH)	*A′*
n.obs.	1346/1344/1343	1312	9.6	ν(CC) +–+–+–	*A′*
1278	1284/1283/1282/1280	1259	38.9	δ(COH), δ(CH) +0++	*A′*
1238/(1245) *^c^*	1240/1239/1235	1217	63.0	ν(CF)	*A′*
1184/(1162) *^c^*	1193/1192/1191/1188	1182	149.7	ν(C–O), δ(COH)	*A′*
1155/1149 *^d^*	1158/1155	1152	26.4	δ(CH) +–+–	*A′*
1111/1105 *^e^*	1116/1115/1113/1110/1106/1103	1105	137.0	ν(C–O), δ(CH) 0++–	*A′*
n.obs.	1093/1091	1086	14.1	ν(C–O), ν(CC) 0+00–0	*A′*
1030/(1059) *^c^*	1032/1031	1031	22.7	δ(ring)_1_	*A′*
973	n.obs.	974	0.6	γ(CH) +–+0	*A″*
952	960	958	1.8	γ(CH) +0–+	*A″*
865	869	865	2.3	γ(CH) ++0–	*A″*
835/(845) *^c^*	841	832	23.6	δ(ring)_2_	*A′*
n.obs.	n.obs.	788	0.002	γ(C=O)	*A″*
755	762/761/760/759/758	756	93.8	γ(CH) ++++	*A″*
744	741/740/739/738/734/733	730	12.2	ν(C–C), δ(ring)_3_	*A′*
696	698/697/696	689	40.8	τ(ring)_1_	*A″*
625	626	623	44.1	δ(OCO)	*A′*
575	587/586/585	576	69.7	τ(C–O)	*A″*
552	538	548	17.3	δ(ring)_3_	*A′*
525	528	531	6.8	δ(ring)_3_, δ(CF)	*A′*
n.obs.	517	516	0.3	τ(ring)_2_, γ(CF)	*A″*
n.i.	429 (?)	425	7.6	γ(C-[CCC]), τ(ring)_3_	*A″*
n.i.	n.i.	369	2.9	δ(ring)_4_	*A′*
n.i.	n.i.	344	0.03	δ(CF)	*A′*
n.i.	n.i.	244	0.7	γ(CF)	*A″*
n.i.	n.i.	208	3.5	γ(COOH)	*A′*
n.i.	n.i.	127	0.8	w(COOH)	*A″*
n.i.	n.i.	28	2.4	τ(C–C)	*A″*

*^a^* Wavenumbers (ν) in cm^−1^, calculated infrared intensities (I ^IR^) in km·mol^−1^; n.obs., not observed; n.i., not investigated; ?, uncertain. *^b^* Approximate description was obtained using the vibrations animation module of Chemcraft [63]; ν, stretching; δ, bending; w, wagging; γ, rocking; τ, torsion; the symbols +, – and 0 placed in front of the ν(CC), ν(CH), δ(CH) and γ(CH) ring coordinates indicate the movement (distending, compressing, staying, respectively) of the related bonds, starting from that located *ortho* to the C=O bond of the carboxylic group and proceeding anti-clockwise through the ring. *^c^* Band due to conformer *cis*-I. *^d^* Fermi interaction with 2 × τ(C-O). *^e^* Fermi interaction with 2 × τ(ring)_3_.

**Table 4:** Experimental (Exp.) and B3LYP/6-311++G(d,p) calculated (Calc.; *cis*-I conformer) infrared spectra of monomeric 2-chlorobenzoic acid, and band assignments *^a^*.

Exp.	Exp. [17]	Calculated		Approximate Description *^b^*	Sym- metry
Gas Phase	Ar Matrix, 9 K	B3LYP			
ν	ν	ν	I ^IR^		
3576	3574/3565	3689	103.6	ν(O–H)	*A*
n.obs.	n.i.	3147	1.7	ν(CH) 00++	*A*
3144	n.i.	3134	2.3	ν(CH) ++00	*A*
3082	n.i.	3121	8.5	ν(CH) +– –+	*A*
3036 (?)	n.i.	3106	2.4	ν(CH) 0+-0	*A*
1767	1766	1760	353.6	ν(C=O)	*A*
1595	1597	1595	33.3	ν(CC) +–++–+	*A*
1571	n.obs.	1569	14.4	ν(CC) +0–+0–	*A*
1477	1481	1467	31.0	ν(CC) +0– –0+, δ(CH) ++– –	*A*
1438	1438	1431	25.4	ν(CC) 0+00–0, δ(CH) +++0	*A*
1334/(1371) *^c^*	1341	1325	85.0	ν(C–O), δ(COH)	*A*
n.obs.	n.obs.	1292	0.2	ν(CC) +–+–+–	*A*
1274	n.obs.	1261	8.8	δ(CH) +00+	*A*
1185	1179/1176	1171	156.9	δ(COH), ν(C-C), δ(CH) 00+0	*A*
~1167	1162	1162	39.8	δ(CH) +–+–	*A*
1134	1143	1134	48.5	δ(CH) 0++–	*A*
1103	1103/1101/1098	1083	85.6	ν(CCl), ν(C–O)	*A*
n.obs.	n.obs.	1041	14.4	δ(ring)_1_	*A*
1045	1048	1029	130.8	δ(ring)_2_	*A*
n.obs.	n.obs.	984	0.2	γ(CH) +–+–	*A*
952	n.obs.	958	2.1	γ(CH) +– –+	*A*
864	n.obs.	867	0.6	γ(CH) ++– –	*A*
800	n.obs.	792	2.2	γ(C=O)	*A*
782/775 *^d^*	n.obs.	775	13.2	ν(C–C), δ(ring)_3_, δ(OCO)	*A*
746	749/747	743	89.6	γ(CH) ++++	*A*
707	686	690	21.8	τ(ring)_1_	*A*
690	n.obs.	677	30.4	ν(CCl), δ(OCO), δ(ring)_3_	*A*
630	631	627	43.7	δ(OCO)	*A*
576	n.obs.	575	78.3	τ(C–O)	*A*
n.i.	n.obs.	526	12.2	γ(C-[CCC])	*A*
n.i.	n.i.	483	2.3	τ(ring)_2_	*A*
n.i.	n.i.	440	3.9	δ(ring)_3_	*A*
n.i.	n.i.	416	6.9	τ(ring)_3_	*A*
n.i.	n.i.	350	1.7	δ(ring)_4_	*A*
n.i.	n.i.	293	0.6	δ(CCl)	*A*
n.i.	n.i.	205	1.8	γ(CCl)	*A*
n.i.	n.i.	192	0.7	γ(COOH)	*A*
n.i.	n.i.	114	0.2	w(COOH)	*A*
n.i.	n.i.	28	0.8	τ(C–C)	*A*

*^a^* Wavenumbers (ν) in cm^−1^, calculated infrared intensities (I^IR^) in km·mol^−1^; n.obs., not observed; n.i., not investigated; ?, uncertain. *^b^* Approximate description was obtained using the vibrations animation module of Chemcraft [63]; ν, stretching; δ, bending; w, wagging; γ, rocking; τ, torsion; the symbols +, – and 0 placed in front of the ν(CC), ν(CH), δ(CH) and γ(CH) ring coordinates indicate the movement (distending, compressing, staying, respectively) of the related bonds, starting from that located *ortho* to the C=O bond of the carboxylic group and proceeding anti-clockwise through the ring. *^c^* Band due to conformer *cis*-II. *^d^* Fermi interaction with τ(C-O) + γ(CCl).

**Table 5:** Experimental (Exp.) and B3LYP/6-311++G(d,p) calculated (Calc.) infrared spectra of monomeric 2,6-difluorobenzoic acid, and band assignments *^a^*.

Exp.	Calculated		Approximate Description *^b^*	Sym- metry
Gas Phase	B3LYP			
ν	ν	I ^IR^		
3570	3670	93.6	ν(O–H)	*A*
n.obs.	3143	0.1	ν(CH) +++	*A*
n.obs	3138	0.7	ν(CH) +0–	*A*
3082	3118	3.5	ν(CH) +–+	*A*
1779	1768	421.1	ν(C=O)	*A*
1621	1623	121.0	ν(CC) +–++–+	*A*
1597/1563 *^c^*	1585	80.2	ν(CC) +0–+0–	*A*
1472	1466	80.1	ν(CC) 0++0– –, δ(CH) 0++	*A*
1472	1461	68.7	ν(CC) ++0– –0, δ(CH) ++0	*A*
1363/1320 *^d^*	1331	115.2	ν(C–O), δ(COH)	*A*
n.obs.	1304	0.3	ν(CC) +–+–+–	*A*
1295	1266	27.4	ν(CF_2_) sym	*A*
1244/1238 *^e^*	1230	65.5	δ(CH) +++	*A*
1182	1179	163.4	δ(COH), ν(C–O)	*A*
1150	1153	0.9	δ(CH) +–+	*A*
1094	1090	109.6	ν(C-O), δ(CH) +0–	*A*
1054	1050	24.9	δ(ring)_1_	*A*
1020	1002	146.0	ν(CF_2_) asym	*A*
964	956	0.5	γ(CH) +–+	*A*
n.obs.	874	0.1	γ(CH) +0–	*A*
798	793	51.2	γ(CH) +++, γ(C=O)	*A*
786	784	10.3	ν(C–C), δ(ring)_2_	*A*
763	763	19.6	γ(C=O), γ(CH) +++	*A*
697	700	44.55	τ(ring)_1_	*A*
683	684	21.7	δ(OCO)	*A*
611 (?)	594	7.0	τ(ring)_3_, γ(CF_2_) asym	*A*
581	588	86.9	τ(C–O)	*A*
n.obs	573	5.2	τ(ring)_2_	*A*
566	554	41.0	δ(OCO), δ(ring)_3_	*A*
n.i.	509	2.4	δ(CF_2_) asym	*A*
n.i.	473	3.0	τ(ring)_3_	*A*
n.i.	400	2.1	δ(ring)_4_	*A*
n.i.	385	1.9	γ(C-[CCC])	*A*
n.i.	283	0.8	δ(CF_2_) sym	*A*
n.i.	236	0.02	γ(CF_2_) asym	*A*
n.i.	218	0.9	γ(CF_2_) sym	*A*
n.i.	165	1.4	w(COOH)	*A*
n.i.	94	2.3	γ(COOH)	*A*
n.i.	44	1.9	τ(C–C)	*A*

*^a^* Wavenumbers (ν) in cm^−1^, calculated infrared intensities (I^IR^) in km·mol^−1^; n.obs., not observed; n.i., not investigated; ?, uncertain. *^b^* Approximate description was obtained using the vibrations animation module of Chemcraft [63]; ν, stretching; δ, bending; w, wagging; γ, rocking; τ, torsion; the symbols +, − and 0 placed in front of the ν(CC), ν(CH), δ(CH) and γ(CH) ring coordinates indicate the movement (distending, compressing, staying, respectively) of the related bonds, starting from that located *ortho* to the C=O bond of the carboxylic group and proceeding anti-clockwise through the ring. *^c^* Fermi interaction with 2 × {γ(CH) +++, γ(C=O)}. *^d^* Fermi interaction with 2 × δ(OCO). *^e^* Fermi interaction with 2 × {τ(ring)_3_, γ(CF_2_) asym}.

**Table 6:** Experimental (Exp.) and B3LYP/6-311++G(d,p) calculated (Calc.) infrared spectra of monomeric 2-chloro-6-fluorobenzoic acid, and band assignments *^a^*.

Exp.	Exp. (This Work; Also [58])	Calc.			Approximate Description *^b^*	Sym- metry
Gas Phase	Xe Matrix, 20 K	B3LYP.				
ν	ν	ν	I ^IR^			
3566	3542/3522/3519/3512/3507	3662	87.5		ν(O–H)	*A*
n.obs.	n.obs.	3141	0.5		ν(CH) +++	*A*
n.obs.	n.obs.	3137	0.4		ν(CH) +0–	*A*
3083	2976	3116	3.6		ν(CH) +–+	*A*
1779	1770	1776	356.3		ν(C=O)	*A*
1602	1611	1607	67.0		ν(CC) +–++–+	*A*
1580	1581/1579	1575	72.6		ν(CC) +0–+0–	*A*
1455	1464	1450	35.2		ν(CC) +0– –0+, δ(CH) +– –	*A*
1455	1457/1454	1446	88.1		ν(CC) ++0– –0, δ(CH) ++0	*A*
1362/1331 *^c^*	1376/1371/1364/1356/1332/1327	1324	96.3		ν(C–O), δ(COH)	*A*
1300	1301/1291	1291	1.5		ν(CC) +–+–+–	*A*
1258	1258/1255	1239	64.7		ν(CF), δ(CH) +++	*A*
1195	1203/1198/1194	1192	70.9		δ(COH), δ(CH) +00	*A*
1175	1178/1173/1172/1169	1167	117.9		ν(C–O), δ(COH), δ(CH) +–+	*A*
1151	1149	1142	26.9		ν(CCl), δ(CH) 0+0	*A*
1093	1101/1098/1096/1094	1093	90.3		ν(C–O), δ(ring)_1_, δ(CH) +0+	*A*
1052	1055/1054/1052	1049	27.7		δ(ring)_2_	*A*
964	n.obs.	956	0.9		γ(CH) +–+	*A*
909	912/910/908/907	887	103.8		ν(CCl), ν(CF)	*A*
n.obs.	n.obs.	880	0.4		γ(CH) +0–	*A*
786	792/791/788/785	778	46.1		γ(CH) +++, γ(C=O)	*A*
779	777	768	11.2		ν(C–C), δ(OCO)	*A*
779	767	760	26.0		γ(C=O), γ(CH) +++	*A*
737	735	715	32.5		τ(ring)_1_	*A*
662	661/659	653	34.0		δ(OCO)	*A*
582	583/580/575/572	591	82.9		τ(C-O)	*A*
n.obs.	n.obs.	565	0.6		τ(ring)_2_	*A*
n.i.	569/567	554	5.2		τ(ring)_3_	*A*
n.i.	545/543/542	538	23.7		δ(ring)_3_	*A*
n.i.	469	463	4.8		δ(ring)_4_, τ(C–O)	*A*
n.i.	n.i.	418	4.4		δ(CFCl) asym	*A*
n.i.	n.i.	388	0.3		γ(C-[CCC])	*A*
n.i.	n.i.	343	1.2		δ(ring)_4_	*A*
n.i.	n.i.	230	0.5		δ(CFCl) sym	*A*
n.i.	n.i.	225	0.3		γ(CF)	*A*
n.i.	n.i.	174	2.0		γ(CCl)	*A*
n.i.	n.i.	141	0.4		w(COOH)	*A*
n.i.	n.i.	85	1.6		γ(COOH)	*A*
n.i.	n.i.	24	2.3		τ(C–C)	*A*

*^a^* Wavenumbers (ν) in cm^−1^, calculated infrared intensities (I^IR^) in km·mol^−1^; n.obs., not observed; n.i., not investigated; ?, uncertain. *^b^* Approximate description was obtained using the vibrations animation module of Chemcraft [63]; ν, stretching; δ, bending; w, wagging; γ, rocking; τ, torsion; the symbols +, – and 0 placed in front of the ν(CC), ν(CH), δ(CH) and γ(CH) ring coordinates indicate the movement (distending, compressing, staying, respectively) of the related bonds, starting from that located *ortho* to the C=O bond of the carboxylic group and proceeding anti-clockwise through the ring. *^c^* Fermi interaction with 2 × δ(OCO).

**Table 7:** Experimental (Exp.) and B3LYP/6-311++G(d,p) calculated (Calc.) infrared spectra of monomeric 2,6-dichlorobenzoic acid, and band assignments *^a^*.

Exp.	Calculated		Approximate	Sym-
Gas Phase	B3LYP		Description *^b^*	metry
ν	ν	I ^IR^		
3564	3660	86.1	ν(O–H)	*A′*
3130	3141	1.0	ν(CH) +++	*A′*
n.obs.	3137	0.1	ν(CH) +0–	*A″*
3073	3114	3.7	ν(CH) +–+	*A′*
1780	1779	330.5	ν(C=O)	*A′*
1587	1587	33.6	ν(CC) +–++–+	*A′*
1567	1563	49.7	ν(CC) +0–+0–	*A″*
1455 (?)	1436	5.2	ν(CC) +0– –0+, δ(CH) +0–	*A′*
1434	1429	79.6	ν(CC) 0+00–0, δ(CH) +++	*A″*
1349/1297 *^c^*	1322	70.9	ν(C–O), δ(COH)	*A′*
1266	1277	2.4	ν(CC) +–+–+–	*A″*
1192	1193	25.0	δ(CH) +0+	*A″*
1192	1189	130.9	δ(COH), δ(ring) _1_	*A′*
1159	1153	7.3	δ(CH) 0+0	*A″*
1127	1120	172.4	ν(C–O), δ(COH), ν(CCl_2_) sym, δ(CH) +0–	*A′*
1090/1083 *^d^*	1073	9.1	ν(CCl_2_) sym	*A′*
1047	1043	24.0	δ(ring)_2_	*A′*
n.obs.	967	1.0	γ(CH) +–+	*A′*
n.obs.	886	0.002	γ(CH) +0–	*A″*
804	785	37.6	ν(CCl_2_) asym, γ(C=O)	*A″*
786	768	26.5	ν(C-C), γ(CH) +++	*A′*
777	766	25.5	γ(CH) +++	*A′*
747/740 *^e^*	746	89.6	γ(C=O), ν(CCl_2_) asym	*A″*
727	729	15.4	τ(ring)_1_	*A′*
650	641	35.8	δ(OCO)	*A′*
582	597	71.7	τ(C-O)	*A″*
n.obs.	567	0.3	τ(ring)_2_	*A*
n.i.	518	0.3	τ(ring)_3_	*A″*
n.i.	454	18.2	ν(C–C), ν(CCl_2_) sym	*A′*
n.i.	432	1.6	δ(ring)_3_	*A″*
n.i.	382	0.3	γ(CCl_2_) sym	*A′*
n.i.	376	2.9	δ(CCl_2_) asym	*A″*
n.i.	313	0.5	δ(ring)_4_	*A′*
n.i.	195	0.001	γ(CCl_2_) asym	*A″*
n.i.	185	0.01	δ(CCl_2_) sym	*A′*
n.i.	164	2.9	γ(C-[CCC])	*A′*
n.i.	135	0.2	w(COOH)	*A″*
n.i.	74	0.8	γ(COOH)	*A′*
n.i.	38	2.1	τ(C–C)	*A″*

*^a^* Wavenumbers (ν) in cm^−1^, calculated infrared intensities (I^IR^) in km·mol^−1^; n.obs., not observed; n.i., not investigated; ?, uncertain. *^b^* Approximate description was obtained using the vibrations animation module of Chemcraft [63]; ν, stretching; δ, bending; w, wagging; γ, rocking; τ, torsion; the symbols +, – and 0 placed in front of the ν(CC), ν(CH), δ(CH) and γ(CH) ring coordinates indicate the movement (distending, compressing, staying, respectively) of the related bonds, starting from that located *ortho* to the C=O bond of the carboxylic group and proceeding anti-clockwise through the ring. *^c^* Fermi interaction with 2 × δ(OCO). *^d^* Fermi interaction with τ(ring)_2_ + τ(ring)_3_. *^e^* Fermi interaction with 2 × δ(CCl_2_) asym.

**Table 8:** Summary of the data for the crystals of the studied compounds *^a^*.

	BA	2FBA	26DFBA	2CBA	26DCBA	2C6FBA
	[30]	[20]	[22]	[21]	[23]	[24]
Crystal system	Monoclinic	Monoclinic	Monoclinic	Monoclinic	Triclinic	Monoclinic
Space group	P2_1_/c	P2_1_/n	P2_1_/c	C2/c	P$\bar{1}$	P2_1_/c
Z	4	4	4	8	4	4
Z′	1	1	1	1	2	1
a/Å	5.4996(5)	6.5523(13)	3.6517(4)	14.6382(16)	7.2678(9)	3.7655(2)
b/Å	5.1283(5)	3.7524(8)	14.1214(15)	3.8142(6)	9.8543(8)	13.9660(7)
c/Å	21.950(2)	25.021(5)	12.2850(13)	23.812(3)	11.8290(11)	13.2300(7)
α/°	90	90	90	90	95.000(7)	90
β/°	97.37(1)	92.82(3)	95.651(3)	101.146(11)	104.262(10)	98.034(3)
γ/°	90	90	90	90	102.128(8)	90
Cell volume/Å^3^	614(1)	614.5(2)	630.42(12)	1304.4(3)	794.07(14)	688.92(6)
Density	1.322	1.515	1.666	1.544	1.598	1.683
Conformer	*cis*	*cis*-I	*cis*	*cis*-I	*cis*	*cis*
φ/°	0.0	10.4(3)	33.7(14)	13.7(2)	88.1//82.2	46.4(2)
Temperature/K	RT	100(2)	100.0(1)	150(2)	198(2)	200(1)

*^a^* Uncertainties are given in parentheses in units of the last digit. RT, room temperature. φ, represents the angle between the planes of the aromatic ring and the carboxylic acid substituent; for 26DCBA the two values presented correspond to the value of this angle in the two non-equivalent molecules in the crystal.

**Table 9:** Atom^…^atom interactions (%) in the crystals of the studied compounds, according to the Hirshfeld method (*d*_norm_ signatures).

Interaction	BA	2FBA	26DFBA	2CBA	26DCBA	2C6FBA
O^…^H	24.9	24.7	23.7	23.9	26.8	23.1
C^…^H	21.1	8.7	5.7	11.1	8.1	6.0
H^…^H	43.0	26.4	12.1	21.7	12.6	13.0
O^…^O	0.9	1.4	0.7	1.1	0.0	1.4
O^…^C	6.4	4.3	4.6	4.3	2.5	3.9
C^…^C	3.6	10.8	11.8	9.4	7.2	10.7
F^…^O		0.0	2.2			1.9
F^…^H		20.0	31.1			11.1
F^…^F		0.2	5.4			1.0
F^…^C		3.4	2.8			0.7
Cl^…^O				0.1	5.9	0.8
Cl^…^H				24.0	24.2	15.8
Cl^…^Cl				1.8	7.3	1.1
Cl^…^C				2.7	5.2	3.4
F^…^Cl						6.3

**Table 10:** CE-B3LYP intreraction energies partition results for the crystals of the studied compounds *^a^*.

Molecule	E_ele	E_pol	E_dis	E_rep	E_tot
BA	−84.4	−25.0	−75.7	110.8	−106.3
2FBA	−81.3	−24.0	−85.6	116.4	−107.0
26DFBA	−87.1	−25.6	−91.9	125.9	−113.8
2CBA	−69.7	−18.6	−87.8	106.6	−98.8
26DCBA	−65.2	−15.3	−75.8	96.8	−87.4
2C6FBA	−82.3	−21.4	−91.4	124.5	−107.0

*^a^* E_ele, E_pol, E-dis, E_rep and E_tot represent the electrostatic, polarization, dispersion, exchange-repulsion, and total interaction energies (in kJ·mol^−1^). E_tot corresponds to the crystal lattice energy (E_lat_) and has been scaled for benchmarked energy models, according to the recommendations [42,43].

**Table 11:** Aromatic ring C-C bond lengths (Å) and HOMA index for the studied molecules (in the crystals and as isolated molecules) *^a^*.

	**Crystal**						
	**BA**	**2FBA**	**2CBA**	**26DFBA**	**26DCBA**	**26DCBA**	**2C6FBA**
					**Mol. 1**	**Mol. 2**	
	1.384	1.401	1.406	1.398	1.388	1.389	1.396
	1.377	1.391	1.385	1.381	1.386	1.381	1.382
	1.380	1.397	1.387	1.387	1.382	1.380	1.377
	1.366	1.394	1.362	1.389	1.378	1.375	1.377
	1.383	1.392	1.408	1.381	1.382	1.387	1.367
	1.386	1.416	1.366	1.396	1.387	1.387	1.389
**HOMA:**	**0.969**	**0.953**	**0.919**	**0.989**	**0.992**	**0.988**	**0.966**
					Average:	**0.990**	
	**Isolated Molecule**						
	**BA**	**2FBA**	**2CBA**	**26DFBA**	**26DCBA**		**2C6FBA**
	1.400	1.402	1.407	1.398	1.397		1.399
	1.390	1.388	1.394	1.385	1.391		1.392
	1.396	1.391	1.390	1.392	1.391		1.392
	1.395	1.395	1.393	1.392	1.391		1.392
	1.392	1.388	1.388	1.385	1.391		1.384
	1.400	1.405	1.405	1.397	1.397		1.393
**HOMA:**	**0.982**	**0.977**	**0.969**	**0.990**	**0.991**		**0.991**

*^a^* C-C bond lengths are presented starting from that connecting the ring carbon atom where the carboxylic acid substituent is bound to the *ortho* carbon atom staying closest to the carbonyl oxygen atom and rotating through the ring. Experimental data for the crystals was taken from Refs. [20,21,22,23,24,30]. Calculated data are from this study and for both 2FBA and 2CBA correspond to the geometrical parameters of the *cis*-I conformer.
